# AMP-Activated Protein Kinase Mediates the Effect of Leptin on Avian Autophagy in a Tissue-Specific Manner

**DOI:** 10.3389/fphys.2018.00541

**Published:** 2018-05-15

**Authors:** Alissa Piekarski, Gurueswar Nagarajan, Peter Ishola, Joshua Flees, Elizabeth S. Greene, Wayne J. Kuenzel, Takeshi Ohkubo, Helena Maier, Walter G. Bottje, Mark A. Cline, Sami Dridi

**Affiliations:** ^1^Center of Excellence for Poultry Science, University of Arkansas, Fayetteville, AR, United States; ^2^College of Agriculture, Ibaraki University, Ibaraki, Japan; ^3^Nidovirus-Cell Interactions Group, The Pirbright Institute, Woking, United Kingdom; ^4^Department of Animal and Poultry Sciences, Virginia Tech, Blacksburg, VA, United States

**Keywords:** leptin, autophagy, AMPK, ICV injection, cell cultures, chickens

## Abstract

Autophagy, a highly conserved intracellular self-digestion process, plays an integral role in maintaining cellular homeostasis. Although emerging evidence indicate that the endocrine system regulates autophagy in mammals, there is still a scarcity of information on autophagy in avian (non-mammalian) species. Here, we show that intracerebroventricular administration of leptin reduces feed intake, modulates the expression of feeding-related hypothalamic neuropeptides, activates leptin receptor and signal transducer and activator of transcription (Ob-Rb/STAT) pathway, and significantly increases the expression of autophagy-related proteins (Atg3, Atg5, Atg7, beclin1, and LC3B) in chicken hypothalamus, liver, and muscle. Similarly, leptin treatment activates Ob-Rb/STAT pathway and increased the expression of autophagy-related markers in chicken hypothalamic organotypic cultures, muscle (QM7) and hepatocyte (Sim-CEL) cell cultures as well as in Chinese Hamster Ovary (CHO-K1) cells-overexpressing chicken Ob-Rb and STAT3. To define the downstream mediator(s) of leptin's effects on autophagy, we determined the role of the master energy sensor AMP-activated protein kinase (AMPK). Leptin treatment significantly increased the phosphorylated levels of AMPKα1/2 at Thr172 site in chicken hypothalamus and liver, but not in muscle. Likewise, AMPKα1/2 was activated by leptin in chicken hypothalamic organotypic culture and Sim-CEL, but not in QM7 cells. Blocking AMPK activity by compound C reverses the autophagy-inducing effect of leptin. Together, these findings indicate that AMPK mediates the effect of leptin on chicken autophagy in a tissue-specific manner.

## Introduction

Autophagy is a highly conserved intracellular self-digestion process by which cells degrade and recycle their own constituents, including damaged organelles, within lysosomes (Levine and Klionsky, 2004; Levine and Kroemer, 2008). There are three types of autophagy; macro-autophagy, micro-autophagy, and chaperone-mediated autophagy (Cuervo, 2011; Boya et al., 2013). Macro- and micro-autophagy involve the engulfment of large structures through both selective (specific organelles such as mitochondria or Golgi apparatus referred to as mitophagy or golgiphagy, respectively) and non-selective mechanisms (bulk cytoplasm), however chaperone-mediated autophagy degrades soluble proteins (Kraft et al., 2008; Orenstein et al., 2013; Baumann, 2015; Khaminets et al., 2015). Unlike the micro-autophagy which is mediated by direct lysosome engulfment of the cytosolic cargo via invagination in their limiting membrane, macro-autophagy (hereafter autophagy) refers to the sequestration within a double-membrane structures called autophagosome which enclose cellular material and fuse with lysosomes (Mortimore et al., 1988; Todde et al., 2009). The autophagic machinery is encoded by several autophagy-related (Atg) genes that orchestrate the different sequential steps of autophagy (Ohsumi, 2014). The first two steps, initiation (step 1) and nucleation (step 2), involve the recruitment of cytosolic components of the core autophagic Atg1 kinase complex (Atg1, Atg13, Atg17, Atg29, and Atg31) to the omegasomes (Mao et al., 2013). These components recruit additional proteins, involving transport protein particle III (TRAPPIII) and Rab1 GTPase which carry coat protein complex II (COPII), Atg9 vesicles, and then the activation of the class III phosphoinositide 3-kinase (PI3k) complex I (Atg6, Atg14, Atg38, vacuolar protein sorting (Vps) 15 and 34), to initiate the expansion and elongation of the double-membrane phagophore (step 3) (Kaufmann et al., 2014; Feng et al., 2016). Activation of PI3K complex I generates the phosphatidylinositol 3-phosphate required to recruit additional autophagic cores including Atg2-Atg18 complex, Atg5-Atg12 conjugation systems (Atg3, Atg4, Atg5, Atg7, Atg10, Atg12, and Atg16), microtubule-associated protein 1 light chain 3 (LC3)-phosphatidylethanolamine (PE) complex, and the ubiquitin-like (UBL) conjugation systems. As a result of this activation, cargo designed for autophagy is surrounded, engulfed, and entrapped into a completed and mature autophagosome (step 4) (Kabeya et al., 2000; Axe et al., 2008; Farre and Subramani, 2016). During the step 5, docking and fusion, autophagosome moves via dynein motor proteins (Ravikumar et al., 2005; Kimura et al., 2008) toward the microtubule organizing center where the lysosomes are enriched. The outer autophagosomal membrane fuses with that of the lysosome in reaction involving several proteins such as ESCRT (Lee et al., 2007), SNAREs (Nair et al., 2011; Itakura et al., 2012), Rab7 (Gutierrez et al., 2004; Jäger et al., 2004), and Vps (Liang et al., 2008) leading, in turn, to the releases of autophagic body into lysosomal lumen for hydrolase degradation and recycling of macromolecular components for cellular use.

Autophagy plays a pivotal role in maintaining cellular metabolism and homeostasis and thereby participates in plethora of (patho)physiological processes ranging from starvation adaptation, intracellular protein and organelle clearance, cell differentiation and development, innate and adaptive immunity, tumor suppression, and lifespan extension (Deretic and Levine, 2009; Pimkina and Murphy, 2009; Hailey et al., 2010; Cinque et al., 2015; Kimmey et al., 2015; Palikaras et al., 2015; Garcia-Prat et al., 2016). Autophagy dysregulation is linked with various diseases such as cancer, metabolic syndrome, cardiomyopathy, muscular disorders, and neurodegeneration (Tanaka et al., 2000; Hara et al., 2006; Singh et al., 2009; Levine et al., 2011; He et al., 2012; Oka et al., 2012; Rosenfeldt et al., 2013). Although the regulation of autophagy is highly complicated, the most typical trigger of autophagy is nutrient (amino acids, nitrogen, and carbon) starvation. Autophagy is also elicited in response to environmental cues such as radiation (Paglin et al., 2001; Jinno-Oue et al., 2010) and reactive oxygen species (Scherz-Shouval et al., 2007). It is now accepted that the endocrine system, particularly insulin (Nemazanyy et al., 2015; Fritzen et al., 2016) and leptin (Malik et al., 2011; Cassano et al., 2014; Słupecka et al., 2014; Nepal et al., 2015) manage autophagy regulation. The autophagy field has explosively expanded and the mass of data are predominantly from studies in mammals and yeast. In contrast, there is a scarcity of information on autophagy in avian (non-mammalian) species. After the characterization of avian autophagy-related genes (Piekarski et al., 2014a), we undertook the current study to determine the effects of leptin and to define its downstream signaling pathway on autophagy machinery in chickens. Because leptin is an anorexigenic hormone and because autophagy plays a key role in cell adaptation to a plethora of stressors including food deprivation (Kuma et al., 2004), we hypothesized that leptin might activate autophagy.

## Materials and methods

### Animals and intracerebroventricular (ICV) injection procedure

Five day post-hatch male Hubbard x Cobb500 chicks were randomly divided into two body-weight (156 ± 5 g average BW) matched groups and assigned to ICV treatment with artificial cerebrospinal fluid (aCSF) as vehicle or leptin treatment (*n* = 10/group). Briefly, recombinant ovine leptin (Protein Laboratory Rehovot, Israel) was dissolved in aCSF as a vehicle for a total injection volume of 5 μL (625 pmol) with 0.1% Evans Blue dye to facilitate injection site localization. Chicks were freehand ICV injected using an adapted method that does not appear to induce physiological stress (Davis et al., 1979). The head of the chick was briefly inserted into a restraining device which approximately orients the skull as described by Kuenzel and Masson (1988). The injection coordinate were 3 mm anterior to the coronal suture, 1 mm lateral from the midline sagittal suture, and 2 mm deep from the external surface of the skull targeting the left lateral ventricle. Anatomical landmarks were determined visually and by palpation. Injection depth was controlled by placing a plastic tubing sheath over the needle. The needle remained at the injection depth in the chick for 5 s post injection to reduce backflow. Feed intake was recorded at 30, 60, and 180 min after injection. After data collection, the birds were euthanized by cervical dislocation and the injection site was verified by brain section made along the frontal plane. Any chick without dye present in the lateral ventricle system was discarded. Hypothalamus, liver, and muscle tissues from dye-positive chicks were harvested and kept at −80°C until use. The dissection of chicken hypothalamus was based on the stereotaxic atlas of the brain of the chick (Kuenzel and Masson, 1988). The hypothalamus defined by the posterior margin of the optic chiasm and the anterior margin of the mammillary bodies to the depth of approximately of 2–4 mm was dissected (Figure S1).

The present study was conducted in accordance with the recommendations in the guide for the care and use of laboratory animals of the National Institutes of Health and the protocols were approved by the University of Arkansas Animal Care and Use Committee under protocol 16084 and the Virginia Tech Institutional Animal Care and Use Committee under protocol 15-236.

### Hypothalamic organotypic culture and treatment

A brain slice culture experiment was performed using 3–5 day old chicks as previously described (Nagarajan et al., 2017). Birds were cervically dislocated, brains were dissected out of the cranium and placed in ice cold aCSF saturated with 95% O_2_ and 5% CO_2_. Brains were then glued using Loctite superglue to a vibratome stage and immediately covered with ice cold aCSF. The vibratome chamber was maintained in an ice cold condition at all time. Brains were sectioned at 350 μm thickness and sections were dissected to contain the posterior mediobasal hypothalamus including the infundibular nucleus, equivalent to the mammalian arcuate nucleus. Slices were then placed on each cell culture insert (Millipore PICM03050) saturated with 1 mL standard culture media (SCM) (50% Eagle basal medium, 25% Hanks Blank salt solution, 25% heat inactivated horse serum, 25U/mL penicillin, 0.5% glucose and 0.5 mM L-glutamine) and maintained at 36°C and 5% CO_2_. The procedure was performed in less than 1 h. Culture media was replaced every 2 or 3 days. Sixteen hours prior to the treatments, SCM was replaced with serum free media (SFM) (100% Eagle basal medium, 25U/ml penicillin, 0.5% glucose and 0.5 mM L-glutamine). Treatments with recombinant ovine leptin alone or in combination with AMPK inhibitors were applied as described below.

### Cell lines and culture conditions

Avian myogenic QM7 (Antin and Ordahl, 1991), hepatocyte Sim-CEL (Piekarski et al., 2014b), and Chinese hamster ovary CHO-K1 cell lines were grown in their appropriate media complemented with 10% FBS (Life Technologies, Waltham, MA) and 1% penicillin-streptomycin (Biobasic, Amherst, NY) at 37°C under a humidified atmosphere of 5% CO_2_ and 95% air, as previously described (Adachi et al., 2008; Piekarski et al., 2014b; Lassiter et al., 2015). At 80–90% confluence, cells were synchronized overnight in serum-free media and treated with leptin alone or in combination with AMPK inhibitors as described below.

### Plasmids preparation and transient transfection

Expression vectors containing the chicken leptin receptor cDNA (pCI-chLEPR called hereafter p-ObR), green fluorescent protein (GFP)-fused chicken signal transducer and activator of transcription 3 cDNA (p-STAT3), and both (p-ObR+STAT3) have been described previously (Adachi et al., 2008; Ohkubo et al., 2014). The backbone vectors pCI-neo and pEGFP-N1 were obtained from Promega (Tokyo, Japan) and were used as controls. QM7, Sim-CEL, and CHO-K1 cells were transiently transfected with p-ObR, p-STAT3, p-ObR+STAT3, or their corresponding backbones using Lipofectamine 2000 (ThermoFisher Scientific, Waltham, MA) according to the manufacturer's protocol. Six hours post transfection, the medium was replaced with serum-free medium and treatments with leptin alone or in combination with AMPK inhibitors were applied.

### Leptin and AMPK inhibitor treatments

Hypothalamic explants, non-transfected and transfected cells (QM7, Sim-CEL, and CHO-K1) were pretreated with compound C (AMPK inhibitor, 20 μM) for 90 min and then treated with recombinant ovine leptin (100 ng/mL, Protein Laboratory Rehovot, Israel) for 24 h. Untreated cells were used as controls. 5-Aminoimidazole-4-carboxamide ribonucleotide (AICAR, 50–250 μM) were used as positive controls.

### RNA isolation, reverse transcription, and real-time quantitative PCR

Total RNA extraction from the hypothalamic explants was conducted as we previously described (Kaneko et al., 2011) with some modifications. Briefly, 24 h after leptin treatment, hypothalamic explants were rinsed twice with ice-cold PBS, and harvested in 1.5 mL lysis buffer (10 mM Tris-HCl (pH 8.0), 140 mM NaCl, 1.5 mM MgCl2, 0.5% Igepal, 2 mM vanadyl ribonuleoside complex (VRC, ThermoFisher Scientific, Waltham, MA)) using bullet blender (Nextadvance, NY). One-tenth of the lysate was added to 1 mL Trizol reagent (ThermoFisher Scientific, Waltham, MA) for total RNA isolation according to manufacturer's recommendations. The rest of the lysate was used for immunoblot as described in the following section. Cell lines were rinsed with cold PBS and harvested directly in Trizol by scraping and gentle pipetting as we previously described (Lassiter et al., 2015). RNA was treated with RQ1 DNAse and reverse transcribed (Quanta Biosciences, Gaithersburg, MD). RNA integrity and quality was evaluated using 1% agarose gel electrophoresis and RNA concentrations and purity were measured for each sample by Take 3 micro volume plate using Synergy HT multi-mode microplate reader (BioTek, Winooski, VT). The RT (cDNA) products were subjected to real-time quantitative PCR (Applied Biosystems 7500 Real-Time PCR system) with SYBR Green Master Mix (ThermoFisher Scientific, Waltham, MA). Oligonucleotide primers used for chicken Atg3, Atg7, Beclin 1, LC3a, LC3b, LC3c, Ob-Rb, STAT1, STAT3, STAT5, and STAT6 are presented in Table 1. Oligonucleotide primers used for chicken feeding-related hypothalamic neuropeptides (NPY, AgRP, POMC, CART, CRH, ORX, and ORXR1/2) as well as the housekeeping gene r18S were previously published (Nguyen et al., 2015; Piekarski et al., 2016). The cycling parameters for the qPCR amplification were as follow: an initial incubation at 50°C for 2 min, an initial denaturation step (95°C, 10 min) followed by 40 cycles of denaturation (95°C, 15 s) and annealing (58°C, 1 min). Melting curve analysis was applied, at the end of the amplification, by using the dissociation protocol (Sequence Detection system) to exclude contamination with unspecific PCR products. The PCR products were also confirmed by 2% agarose gel which exhibit only one definite band of the predicted size and by sequencing. There was no gel-detected bands for the negative controls where the RT products were omitted. Relative expressions of target genes were determined by the 2^−ΔΔCt^ method (Schmittgen and Livak, 2008).

**Table 1:** Oligonucleotide real-time qPCR primers.

**Gene**	**Accession number^a^**	**Primer sequence (5^′^ → 3^′^)**	**Orientation**	**Product size (bp)**
Atg3	NM_001278070	GAACGTCATCAACACGGTGAA TGAGGACGGGAGTGAGGTACTC	Forward Reverse	65
Atg7	NM_001030592	CGATGAACCCAAAAGGTCAGA ACTGGCAATGCGTGTTTCAG	Forward Reverse	57
Beclin 1	NM_001006332	TGCATGCCCTTGCTAACAAA CCATACGGTACAAGACGGTATCTTT	Forward Reverse	61
LC3a	XM_417327	CCTTGTCCCAGACCATGTCAA AGCGGCGCCGGATT	Forward Reverse	56
LC3b	NM_001031461	AACTCCAACCAGGCCTTCTTC GTGGAGACGCTCACCATGCT	Forward Reverse	59
LC3c	XM_419549	CCTGTTGGACAAAACCAAGTTTC ATGGTGATGAACTGCGTCATG	Forward Reverse	63
Ob-Rb	NM_204323	GCAAGACCCTCTCCCTTATCTCT TCTGTGAAAGCATCATCCTGATCT	Forward Reverse	70
STAT1	NM_001012914	AGTTCACCAGCTGTACGATGACA TCCAGCCACTGTGCCAAGTA	Forward Reverse	66
STAT3	AY641397	GCATGTCGTTTGCGGAAAT CAGAATGTTGGTGGCATCCA	Forward Reverse	59
STAT5	AF074248	TCACCATCGCGTGGAAGTT GGTGGTGAAAGGCATCAGGTT	Forward Reverse	65
STAT6	XM_003643566	AGGCAGCTGCGCAACCT ATCCGAACGCCTGATCCTT	Forward Reverse	52

a *Accession number refer to Genbank (NCBI)*.

*Atg3 and 7, autophagy related gene; LC3a, b, and c, microtubule associated protein 1 light chain 3; Ob-Rb, leptin receptor; STAT, signal transducer and activator of transcription*.

### Western blot

Total proteins were quantified and subjected to Western blot as we previously described (Kaneko et al., 2011; Dridi et al., 2012; Nguyen et al., 2015). The rabbit polyclonal anti-Atg3 (#3415), anti-Atg5 (#12994), anti-Atg7 (#8558), anti-beclin 1(#3495), anti-LC3B (#3868), anti-Ob-Rb (#sc-8325), anti-phospho STAT3^Tyr705^ (#9145), anti-STAT3 (#4904), anti-phospho AMP-activated protein kinase alpha (AMPKα1/2)^Thr172^ (#2531), and anti-AMPKα1/2 (#2795) were used at 1:1,000 dilution. To avoid any inter-assay variability, the immunoblots were performed using the same membrane. After each immunoblot, the membrane was stripped and verified for the absence of signal and re-probed with the next antibody. At the end, protein loading was assessed by re-immunoblotting with the use of rabbit anti-β actin (#4967) or anti-GAPDH (#sc-25778) as housekeeping proteins. Pre-stained molecular weight marker (Precision Plus Protein Dual Color) was used as a standard (BioRad, Hercules, CA) and as indicator for transfer efficiency. All primary antibodies were purchased from Cell Signaling Technology (Danvers, MA), except for the anti-Ob-Rb and anti-GAPDH which were purchased from Santa Cruz Biotechnology (Dallas, TX). The secondary antibodies were used (1:5,000) for 1 h at room temperature. The signal was visualized by enhanced chemiluminescence (ECL plus) (GE Healthcare Bio-Sciences, Buckinghamshire, UK) and captured by FluorChem M MultiFluor System (Proteinsimple, Santa Clara, CA). Image Acquisition and Analysis were performed by AlphaView software (Version 3.4.0, 1993-2011, Proteinsimple, Santa Clara, CA).

### Immunofluorescence staining

Immunofluorescence was conducted as previously described (Dridi et al., 2012). Briefly, Sim-CEL, QM7, or CHO-K1 cells were cultured to 50–60% confluency in chamber slides (Lab-Tek, Hatfield, PA) and fixed in methanol for 10 min at −20°C. Cells were blocked with protein block serum free blocking buffer (Dako, Carpinteria, CA) and incubated with rabbit anti-Atg3, anti-Atg5, anti-Atg7, anti-beclin1, anti-LC3B, anti-Ob-Rb, or anti-pSTAT3^Tyr705^ antibody (1:200) overnight at 4°C and visualized with Alexa Fluor 488 or 594-conjugated secondary antibody (Molecular probes, Life Technologies, grand Island, NY). After DAPI counterstaining, slides were cover slipped in Vectashield (Vector Laboratories, Burlingame, CA). Images were obtained using the Zeiss Imager M2. All analysis was performed using AxioVision SE64 4.9.1 SP1 software (Carl Zeiss Microscopy GmbH 2006-2013). The intensity of phospho-STAT3 was normalized to that of STAT3, and the ratio was presented after normalization to 1 in the control group and adjustment for the treated group.

### Statistical analyses

Data were analyzed by one-way analysis of variance (ANOVA). Significant differences among individual group means were determined with Student *t*-test or Student Newman Keuls *post hoc* test using the Graph Pad Prism version 6.00 for Windows, Graph Pad Software (La Jolla, CA). Significance was set at *P* < 0.05. Data are expressed as the mean ± SEM.

## Results

### ICV-leptin administration reduced feed intake and modulated the expression of feeding-related hypothalamic neuropeptides in chickens

**Figure 1:**
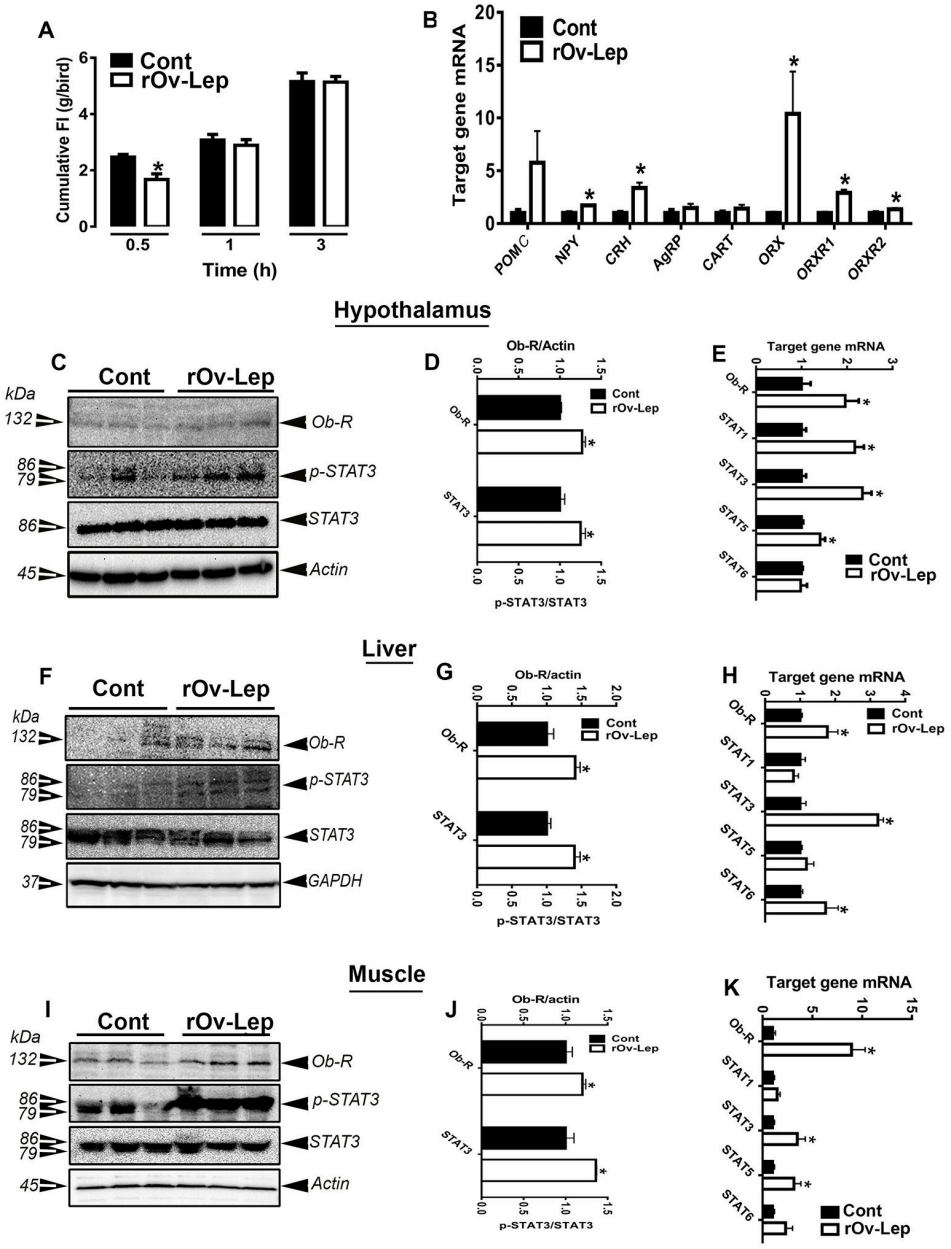
Effect of ICV injection of leptin on feed intake and hypothalamic neuropeptides and Ob-Rb/STAT3 pathway in chickens. Feed intake was recorded at 30, 60, and 180 min after ICV administration of recombinant ovine leptin (625 pmol/10 μL/bird) **(A)**. The relative expression of feeding-related hypothalamic neuropeptides was determined by real-time qPCR **(B)**. Protein levels of Ob-Rb and p-STAT3 and pan STAT3 were measured using Western blot **(C,F,I)** and their relative expression was presented as normalized ratio to pan STAT3 or housekeeping protein (actin or GAPDH) **(D,G,J)**. mRNA abundances of Ob-Rb and STAT genes were measured by qPCR **(E,H,K)**. Data are presented as mean ± SEM (*n* = 10/group). ^*^Indicates significant difference at *P* < 0.05. Cont, control; rOv-Lep, recombinant ovine leptin.

As shown in Figure 1A, leptin significantly reduced feed intake compared to the placebo (aCSF)-treated group at 30 min post administration. However, there was no significant effect of the hormone on feed intake at 60 and 180 min post treatment. Similarly, there was no significant difference in body weights between the two groups (158.3 ± 3.25 vs. 153.5 ± 1.75 for control and leptin-treated group, respectively). The hypothalamic expression of neuropeptide Y (NPY), corticotropin releasing hormone (CRH), orexin (ORX) and its related receptors ORXR1 and ORXR2 was significantly upregulated in leptin-treated compared to the control group at 180 min post treatment (Figure 1B). However, the expression of proopiomelanocortin (POMC), agouti-related protein (AgRP), and cocaine-and amphetamine-regulated transcript (CART) did not differ between the two groups (Figure 1B).

### ICV leptin administration activated Ob-Rb and STAT signaling cascade in chicken tissues

ICV leptin administration significantly upregulated the expression of the long leptin receptor isoform (mRNA and protein) in the chicken hypothalamus (Figures 1C,D), liver (Figures 1F,G), and muscle (Figures 1I,J). Similarly, central leptin administration significantly increased mRNA abundances of STAT1, STAT3, STAT5, but not STAT6 in the hypothalamus (Figure 1E), STAT3 and STAT6 in the liver (Figure 1H), and STAT3 and STAT5 in the muscle (Figure 1K). Central leptin treatment induced STAT3 tyrosine 705 phosphorylation in all three studied tissues (Figures 1C,D,F,G,I,J).

### ICV leptin administration induced autophagy in chicken tissues

**Figure 2:**
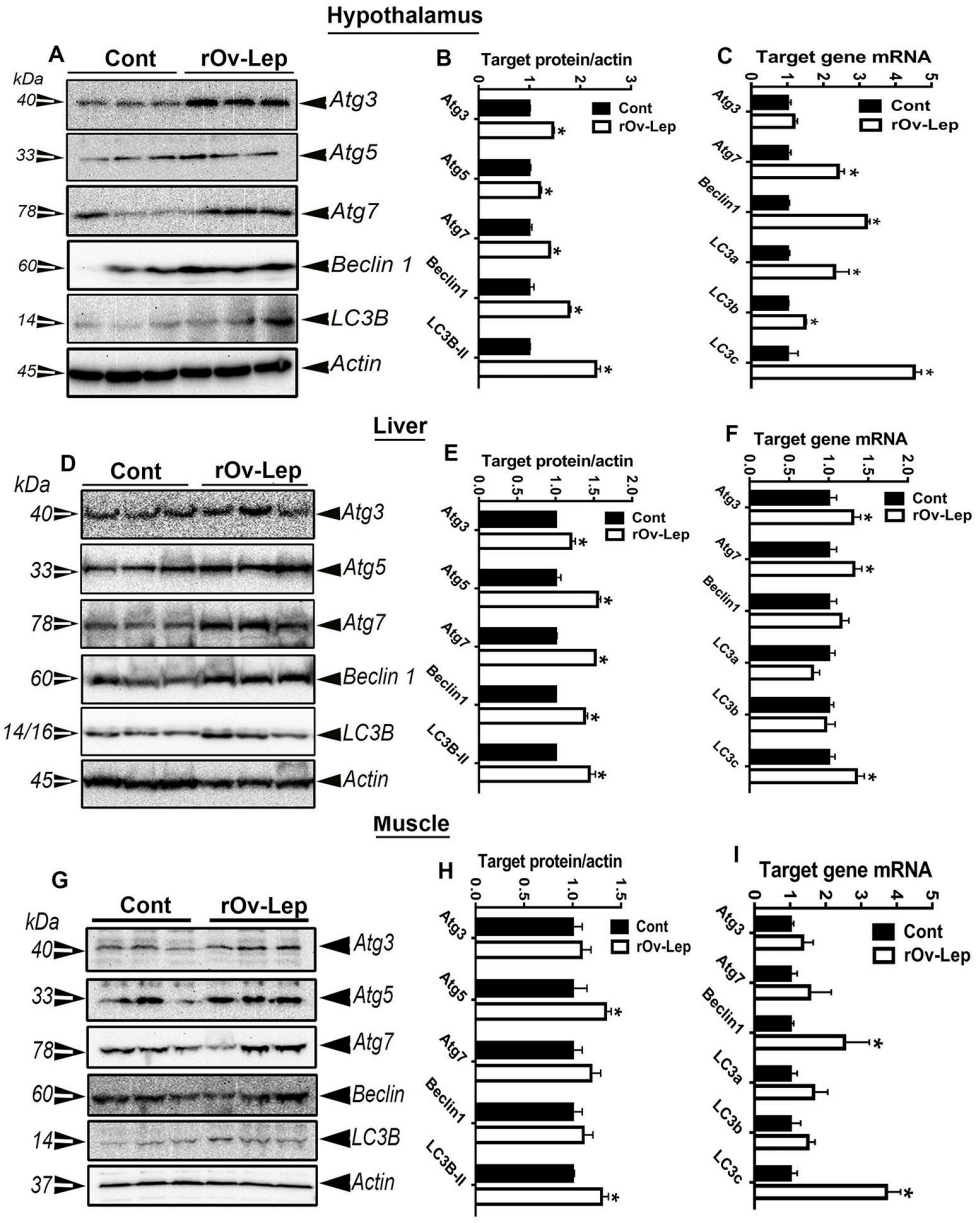
Effect of ICV injection of leptin on autophagy in chicken tissues. Protein levels of autophagy-related markers (Atg3, Atg5, Atg7, beclin1, and LC3B) in hypothalamus **(A)**, liver **(D)**, and muscle **(G)** were determined by Western blot and their relative expression was presented as normalized ratio to housekeeping actin protein **(B,E,H)**. Their mRNA abundances were measured by qPCR **(C,F,I)**. Data are presented as mean ± SEM (*n* = 10/group). ^*^Indicates significant difference at *P* < 0.05.

The protein levels of key autophagy-related markers (Atg3, Atg5, Atg7, Beclin1, and LC3B) were significantly increased in the hypothalamus and liver of leptin-treated compared to control groups (Figures 2A–E). In the muscle, however, only Atg5 and LC3B protein expression was significantly induced by leptin treatment compared to the placebo (Figures 2G,H). Quantitative real-time PCR analyses showed that the mRNA abundances of hypothalamic Atg7, beclin1, LC3a, LC3b and LC3c, hepatic Atg3, Atg7, and LC3c, as well as the muscle beclin1 and LC3c were significantly increased in leptin-treated compared to control groups (Figures 2C,F,I).

### Leptin treatment activated Ob-Rb and STAT signaling pathway in avian hypothalamic explants and Sim-CEL/QM7 cell lines

**Figure 3:**
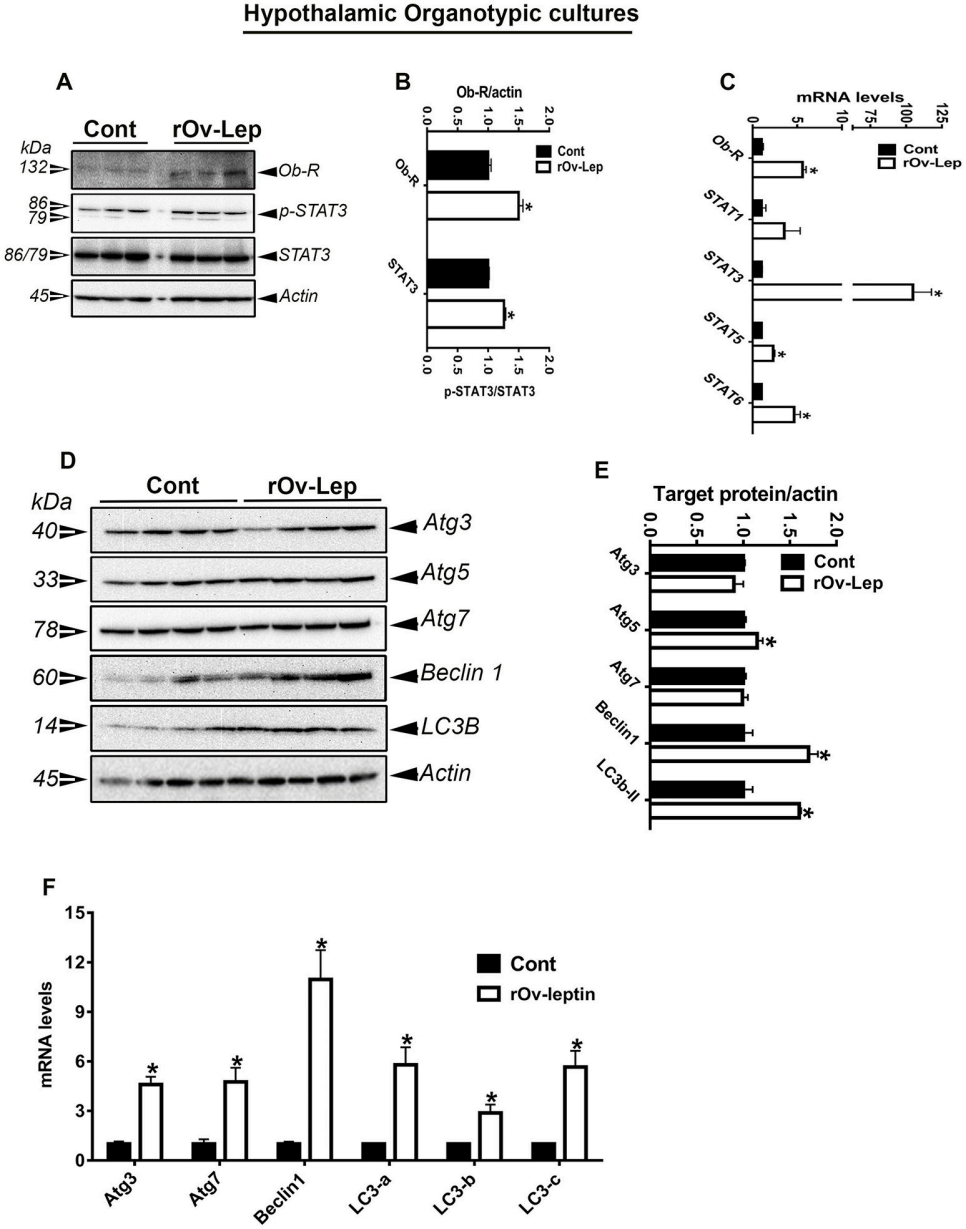
Effect of leptin treatment on Ob-Rb/STAT pathway and autophagy in chicken hypothalamic organotypic culture. Chicken hypothalamic explants were treated with recombinant ovine leptin (100 ng/mL) for 24 h and protein levels of Ob-Rb, STAT3, and autophagy pathways were measured by Western blot **(A,D)**. Their relative expression was presented as normalized ratio to pan STAT3 or actin **(B,E)**. Their mRNA abundances were measured by qPCR **(C,F)**. Data are presented as mean ± SEM (representative of 3 experiments, *n* = 9). ^*^Indicates significant difference at *P* < 0.05.

**Figure 4:**
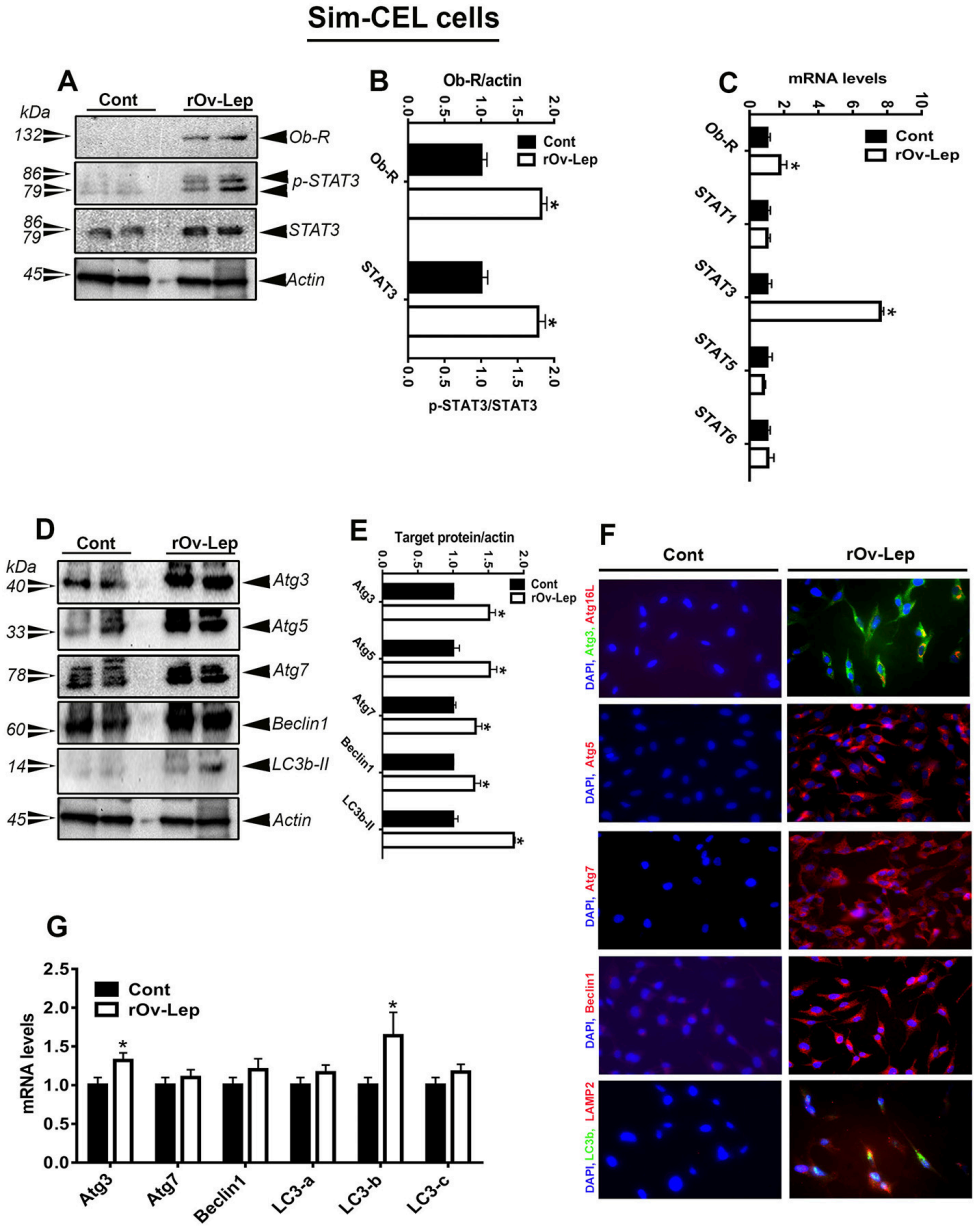
Effect of leptin treatment on Ob-Rb/STAT pathway and autophagy in spontaneously immortalized chicken embryo liver (Sim-CEL) cells. Sim-CEL cells were treated with recombinant ovine leptin (100 ng/mL) for 24 h and protein levels of Ob-Rb, STAT3, and autophagy pathways were measured by Western blot **(A,D)**. Their relative expression was presented as normalized ratio to pan STAT3 or actin **(B,E)**. Their mRNA abundances were measured by qPCR **(C,G)**. Autophagosome and autolysosome formation as well as autophagy-related protein abundance were determined also by immunofluorescence **(F)**. Data are presented as mean ± SEM (representative of 3 experiments, *n* = 6). ^*^Indicates significant difference at *P* < 0.05.

**Figure 5:**
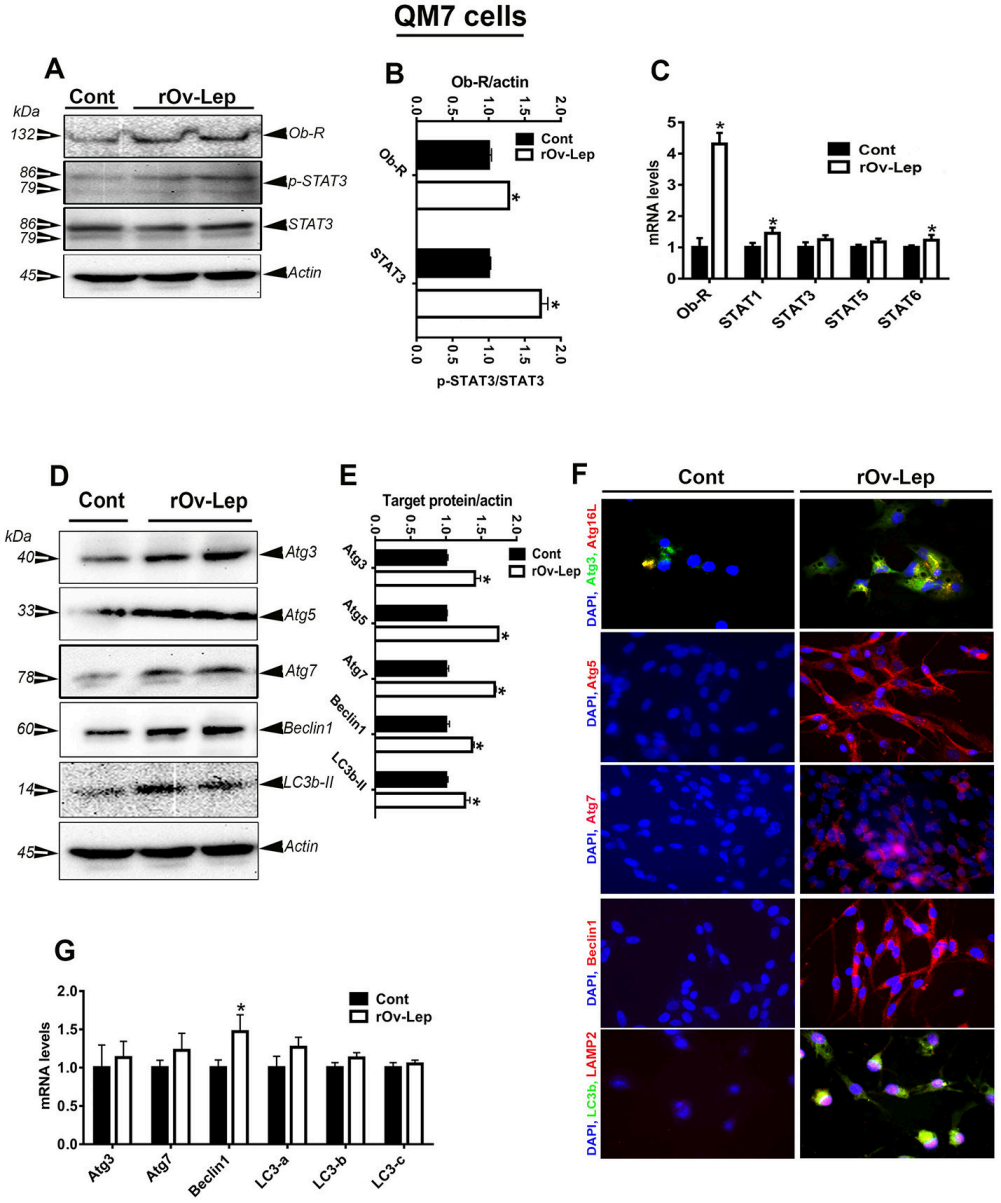
Effect of leptin treatment on Ob-Rb/STAT pathway and autophagy in avian muscle (QM7) cells. QM7 cells were treated with recombinant ovine leptin (100 ng/mL) for 24 h and protein levels of Ob-Rb, STAT3, and autophagy pathways were measured by Western blot **(A,D)**. Their relative expression was presented as normalized ratio to pan STAT3 or actin **(B,E)**. Their mRNA abundances were measured by qPCR **(C,G)**. Autophagosome and autolysosome formation as well as autophagy-related protein abundance were determined also by immunofluorescence **(F)**. Data are presented as mean ± SEM (representative of 3 experiments, *n* = 6). ^*^Indicates significant difference at *P* < 0.05.

To determine whether the effect of leptin was direct or not, chicken hypothalamic explants as well as avian liver (Sim-CEL) and muscle (QM7) cell lines were cultured and treated *in vitro* with recombinant ovine leptin for 24 h. As shown in Figures 3A–C, 4A–C, 5A–C, and in line with *in vivo* data, leptin treatment activated Ob-Rb and STAT signaling pathway in chicken hypothalamic explants and avian Sim-CEL and QM7 cells lines. Indeed, leptin administration significantly increased Ob-Rb protein levels and induced STAT3 tyrosine 705-phosphorylation in the chicken hypothalamic explants, Sim-CEL and QM7 cells (Figures 3A,B, 4A,B, 5A,**B**). Real-time PCR analyses showed that leptin treatment upregulated the expression of STAT3, STAT5, and STAT6 genes in the hypothalamic explants (Figure 3C), STAT3 in Sim-CEL cells (Figure 4C), and STAT1 and STAT6 in QM7 cells (Figure 5C).

### Leptin treatment induced autophagy in avian hypothalamic explants and Sim-CEL/QM7 cell lines

Leptin treatment increased Atg3, Atg7, beclin1, LC3a, LC3b, and LC3c mRNA levels in chicken hypothalamic explants (Figure 3F), Atg3 and LC3b in Sim-CEL cells (Figure 4G), and beclin1 in QM7 cells (Figure 5G). Western blot analyses showed that Atg5, beclin1, and LC3B protein expression was significantly upregulated in leptin-treated compared to untreated chicken hypothalamic explants (Figures 3D,E). In leptin-treated Sim-CEL and QM7 cells, however, the expression of all studied autophagy-related markers (Atg3, Atg5, Atg7, beclin1, and LC3B) was significantly increased compared to untreated cells (Figures 4D,E, 5D,E). The upregulated expression of these autophagy-related proteins along with Atg16L and the lysosome/endosome marker LAMP2 was confirmed by immunofluorescence staining in Sim-CEL and QM7 cell lines (Figures 4F, 5F, respectively).

### Leptin treatment induced autophagy in CHO-K1 cells transfected with chicken OB-Rb and STAT3

**Figure 6:**
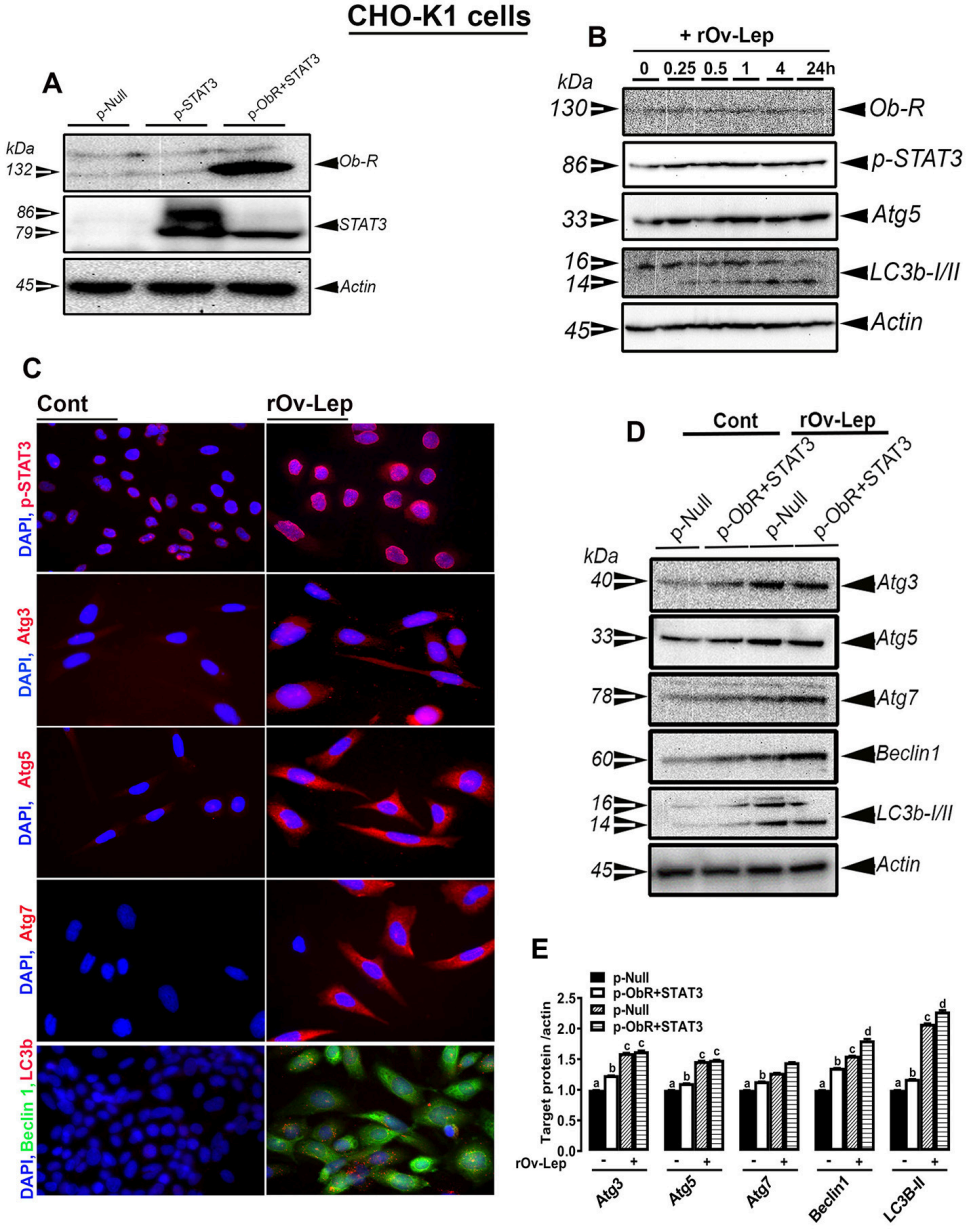
Effect of leptin treatment on Ob-Rb/STAT pathway and autophagy in CHO-K1 cells overexpressing chicken Ob-Rb and STAT3. CHO-K1 cells were efficiently co-transfected with chicken Ob-Rb and STAT3 **(A)**. Leptin treatment (100 ng/mL) induces Ob-Rb, STAT3, Atg5, and LC3B in a time-dependent manner **(B)**. Leptin treatment for 24 h activates autophagy markers in CHO-K1 cells overexpressing chicken Ob-Rb and STAT3 as illustrated by Western blot analysis **(D,E)**. Representative immunofluorescence staining showed a nuclear translocation of p-STAT3 and abundance of autophagy-related proteins **(C)**. Data are presented as mean ± SEM (representative of 3 experiments, *n* = 4). ^*^Indicates significant difference at *P* < 0.05.

As depicted in Figure 6A, CHO-K1 cells were efficiently transfected with chicken Ob-Rb and STAT3. The time course study showed that leptin treatment induced STAT3 phosphorylation at Y^705^ site in CHO-K1 cells transfected with chicken Ob-R/STAT3 and this activation occurred as early as 15 min post-treatment and was maintained for 24 h (Figure 6B). The time course induction of STAT3 coincided with that of Atg5 and LC3B (Figure 6B). Immunofluorescence staining showed that leptin treatment for 24 h activated the nuclear translocation of phosphorylated (Y^705^) STAT3 and upregulated the expression of Atg3, Atg5, Atg7, beclin1, and LC3B (Figure 6C). The upregulation of these autophagy-related markers following 24 h-leptin treatment was confirmed by Western blot analyses (Figures 6D,E). Similarly, chicken OB-Rb and STAT3 plasmid was successfully overexpressed in avian Sim-CEL and QM7 cells (Figures S2, S3). As for CHO-K1 cells, leptin treatment intensified further the increased expression of autophagy-related markers (Atg3, Atg5, Atg7, Beclin1, and LC3B) in Sim-CEL and QM7 cells (Figures 1, 2).

### AMPK mediates leptin's effects on avian autophagy activation in a tissue-specific manner

**Figure 7:**
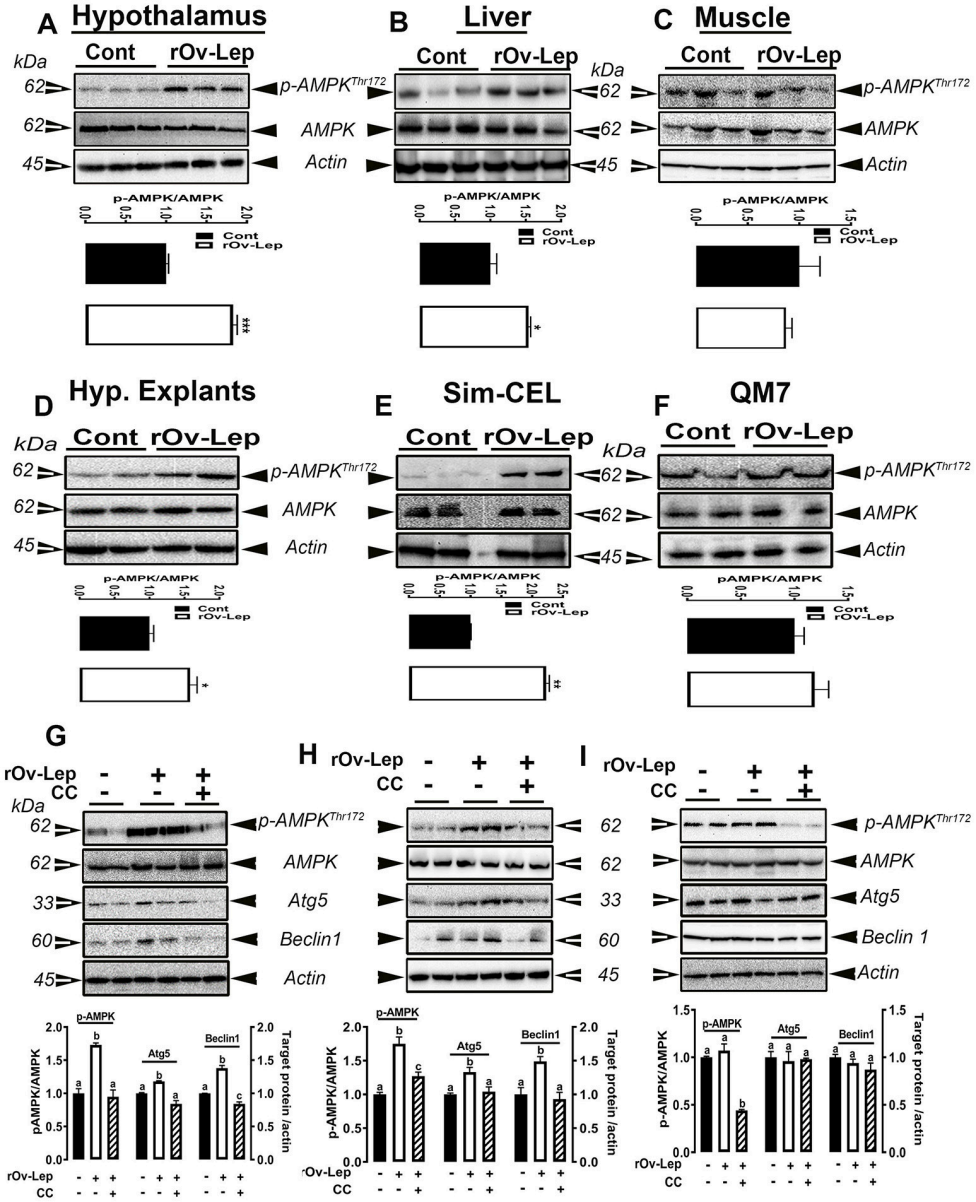
Effect of AMPK blockade on autophagy-induced by leptin. ICV leptin administration activates AMPK in chicken hypothalamus **(A)**, liver **(B)**, and muscle **(C)**. Leptin treatment also induces AMPK activation in chicken hypothalamic explants **(D)**, Sim-CEL **(E)**, and QM7 **(F)** cells. Blocking AMPK activity by compound C attenuates leptin-induced autophagy only in hypothalamic organotypic cultures **(G)** and Sim-CEL **(H)**, but not in QM7 cells **(I)**. Data are presented as mean ± SEM (*n* = 10 for the *in vivo* study, and *n* = 4–6 for the *in vitro* study). ^*^And different letters indicates significant difference at *P* < 0.05. CC, compound C.

ICV leptin administration significantly increased AMPKα1/2 phosphorylation at Thr^172^ site in chicken hypothalamus and liver, but not in muscle (Figures 7A–C). Consistent with these data, *in vitro* studies showed that leptin treatment for 24 h also activated AMPKα1/2 (Thr^172^) in chicken hypothalamic organotypic culture and liver Sim-CEL cells, but not in muscle QM7 cells (Figures 7D–F). Pre-treatment with compound C inhibited AMPK activation in hypothalamic organotypic cultures, Sim-CEL and QM7 cells and attenuated leptin-induced autophagy only in hypothalamic organotypic cultures and Sim-CEL, but not in QM7 cells (Figures 7G–I). AICAR treatment alone for 24 h activated AMPK in avian hypothalamic organotypic culture, Sim-CEL and QM7 cells in a dose-dependent manner (Figures S4A–F). This AMPK activation was accompanied by a significant increase in autophagy-related markers (Atg5 and beclin1) only in the hypothalamic organotypic cultures but not in Sim-CEL or QM7 cells (Figures S4A–F).

## Discussion

This is the first report showing regulation of autophagy by leptin in avian (non-mammalian) species. Leptin is an adipocytokine that is primarily produced by mammalian white adipocytes (Zhang et al., 1994), although it is also expressed in several other tissues including the stomach (Bado et al., 1998), lungs (Vernooy et al., 2009), placenta (Hoggard et al., 1997a), and brain (Wiesner et al., 1999). Leptin is secreted into the blood stream in proportion to the size of white fat depots, and transported across the blood-brain barrier where it binds to its receptor and functions as a negative feedback signal in the regulation of energy balance.

There are multiple isoforms of the leptin receptors that have different length of the intracellular domain. The full-length isoform, (Ob-Rb), is the only receptor that contains intracellular motifs required for activation of the Janus kinase (JAK) and signal transducer and activator of transcription (STATs) signal transduction pathway, and is considered to be the functional receptor. Leptin receptors are ubiquitously expressed in many tissues including hypothalamus (Scott et al., 2009), liver, lung, spleen, kidney, adrenal (Hoggard et al., 1997b), adipocyte (Kielar et al., 1998), immune cells (Lord et al., 1998; Agrawal et al., 2011), and reproductive tissues (Zamorano et al., 1997). Together, the wide expression of leptin and its related receptors indicate that leptin plays myriad physiological roles. Beyond its central function as an anorexigenic and satiety factor, leptin has many pleiotropic effects in different physiological systems such as nutrient intestinal absorption, angiogenesis, lipolysis and lipogenesis, growth, reproduction, inflammation, and immunity (Friedman and Halaas, 1998).

Although the sequence of chicken leptin gene was a matter of intense debate for almost 20 years (Taouis et al., 1998; Ashwell et al., 1999; Seroussi et al., 2016), the chicken leptin receptor sequence however was found to have at least 60% homology with the mammalian orthologs with highly conserved motifs in the transmembrane domain (Horev et al., 2000; Ohkubo et al., 2000). Moreover, previous *in vitro* and *in vivo* studies have shown that chicken leptin receptor is responsive to recombinant mammalian (mouse, human, and ovine) leptins (Denbow et al., 2000; Dridi et al., 2000; Lohmus et al., 2003; Lõhmus et al., 2004; Manjunathan and Ragunathan, 2015). As the recombinant protein for the newly discovered chicken leptin (Seroussi et al., 2016) was not available at the time we undertook this study, we determined whether recombinant ovine leptin can modulate the autophagy pathway *in vivo* following ICV administration to broiler chickens. In agreement with previous studies (Denbow et al., 2000; Dridi et al., 2000; Lohmus et al., 2003), we have firstly shown that leptin administration reduced feed intake at 30 min post-treatment which indicated that the recombinant ovine leptin was biologically active. Secondly and as expected, ICV-injected leptin upregulated the expression of Atg3, Atg5, Atg7, Beclin 1, and LC3B in both the hypothalamus and liver, and only Atg5 and LC3B proteins in the muscle. This suggests that the regulation of autophagy-related proteins by leptin is tissue-specific (Tracy et al., 2016). This differential regulation might be associated with a non-uniform rate of protein turnover between tissues (Hamer et al., 2010). Furthermore, it might be that the spatio-temporal pattern of autophagic-multistep process varied substantially between tissues. For instance, the upregulation of Atg5 suggested an induction of the phagophore formation step in the muscle, however the upregulation of Atg3 and Atg7 indicated an LC3 lipidation and autophagocytosis process in the hypothalamus and liver. The upregulation of Beclin 1 expression, along with Ulk-1 (data not shown), in chicken liver and hypothalamus argues that leptin might recruit an additional pathway for autophagy activation that warrants further in-depth investigation (Kundu et al., 2008; Nishida et al., 2009). Although studies in mammals reported different effects (up or downregulation) of leptin on autophagy (Malik et al., 2011; Cassano et al., 2014; Słupecka et al., 2014), the overall increase in the three paralogs of avian LC3 (LC3a, b, and c) levels indicated that leptin induce autophagosome formation (Maier et al., 2013), which is a key readout of autophagy levels (Barth et al., 2010; Klionsky et al., 2016), in all studied chicken tissues.

Interestingly, the effect of leptin on autophagy activation seemed to be feed intake-independent because there was no significant difference in energy intake between the control and leptin-treated groups at the time of tissue collection (3 h post-treatment), suggesting that leptin might have a direct effect (Cassano et al., 2014). To test this hypothesis and in attempt to mechanistically define the downstream signaling pathways that mediate the effect of leptin on autophagy activation, we used chicken hypothalamic organotypic cultures as well as avian hepatocyte (Sim-CEL) and myoblast (QM7) cell lines. Similarly to the *in vivo* study, we found that leptin administration regulated the expression of autophagy-related proteins in a cell-specific manner; upregulation of Atg3, Atg5, Atg7, Beclin1, and LC3B in Sim-CEL and QM7 cells, and only Atg5, Beclin1, and LC3B in the hypothalamic explants. The upregulation of LC3B expression indicated that leptin activates autophagy in all *in vitro* (hypothalamic, Sim-CEL, and QM7) tissue cultures. The absence of correlation between mRNA abundances and protein levels of some autophagy-related markers observed in both *in vivo* and *in vitro* studies suggested that these proteins are regulated at translational and posttranslational levels (Feng et al., 2015; Xie et al., 2015).

To gain further insights in the mode of leptin action, we next determined the expression profile of Ob-Rb and STAT tyrosine kinase. Leptin treatment upregulated the expression of Ob-Rb at mRNA and protein levels in hypothalamic organotypic cultures, Sim-CEL and QM7 cells. However, the regulation of STAT gene expressions by leptin appear to be cell- and gene-specific. Indeed, leptin treatment increased STAT3, STAT5, and STAT6 mRNA abundances in chicken hypothalamic organotypic cultures, STAT3 in Sim-CEL cells, and STAT1 and STAT6 in QM7 cells. This result is not surprising since leptin has been previously shown to differentially regulate the expression of STAT genes (Sweeney, 2002) which might be due to differential ligand-receptor binding affinities. As STAT3 phosphorylation is a well-established mechanism mediating many of leptin's actions (Vaisse et al., 1996; Niswender et al., 2001), we subsequently determined the phospho (Tyr705)-STAT3 levels and found that leptin treatment activated STAT3 in all tested tissue cultures. Upon activation by leptin, STAT3 has been shown to dimerize and translocate to the nucleus where it regulates transcriptional activity of its target genes (Ihle, 1995). Next, we co-overexpressed chicken Ob-Rb and chicken STAT3 in Chinese hamster ovary (CHO-K1) cells and demonstrated that leptin administration activated Ob-Rb/STAT3 signaling pathway and induced autophagy-related protein expression. In support of these data, immunofluorescence staining showed a nuclear translocation of p-chicken STAT3 in CHO-K1 cells and LC3B puncta confirming again active autophagy and autophagosome formation upon leptin treatment. Similarly, the effect of leptin on autophagy was intensified in Sim-CEl and QM7 cells overexpressing chicken leptin receptor and STAT3 (Supplementary Figures). In line with these *in vitro* studies, we found that ICV- injected leptin signaled also via Ob-Rb and STAT3 pathway. These results confirm again that chicken leptin receptor is responsive to recombinant mammalian leptin (Adachi et al., 2008). Even with this compelling evidence we cannot rule out the involvement of other STATs and/or more than one intracellular signal transduction pathway in mediating the effect of leptin (Takahashi et al., 1997; Cui et al., 2006)

As autophagy is tightly linked to the intracellular energy status, we sought to determine whether the master energy sensor, AMPK (Hardie, 2007), plays a role in mediating leptin's effects. Central administration of leptin induced AMPKα1/2 phosphorylation at Thr172 site in both chicken hypothalamus and liver, but not in muscle. Comparable effects were also observed in the *in vitro* studies where AMPKα1/2 was activated in the hypothalamic explants and Sim-CEL, but not in QM7 cells following leptin treatment. Mechanistically, the effects of leptin on hypothalamic and hepatic autophagy activation were suppressed *in vitro* by inhibition of AMPK with compound C. These results indicate firstly that leptin has a tissue-specific effect on AMPK in chickens as previously shown in mammals but in an opposite direction (Minokoshi et al., 2002, 2004; Andersson et al., 2004). Secondly, these data suggest that the effect of leptin on muscle autophagy might be mediated through other signaling pathways independently of AMPK. Indeed, leptin was found to modulate Ca^2+^ signaling (Tudurí et al., 2013) and an AMPK-independent pathway triggers Ca^2+^-mediated autophagy has been recently reported (Grotemeier et al., 2010). Moreover, leptin has been shown to induce autophagy via the tumor suppressor P53/forkhead box O3A (FoxO3A) axis (Fiorini et al., 2013). The autophagic program has also been shown to be activated through additional complex signal transduction pathways that involve the intertwined activation of mitogen-activated protein kinases (Wei et al., 2008), dual-specific protein phosphatases (DUSPs) (Wang et al., 2016), nuclear factor-kB (NF-kB) (Criollo et al., 2010), and Sirtuin1 (Morselli et al., 2010) to mention a few. All these pathways have been found to interact with leptin (Sun et al., 2013; Marwarha et al., 2014). Even though much of what has been learned about the downstream targets of AMPK has come from the use of AICAR, it is worth noting that in our experimental conditions, the activation of AMPK by AICAR did not elicit any change in the expression of autophagy-related proteins in Sim-CEL, or QM7 cells (Figure S3). This differential effects between leptin and AICAR on the autophagic program activation might be due to the recruitment of different AMPK-downstream mediators. Such scenarios were previously observed in rat insulinoma INS-1E cells where both AICAR and Metformin phosphorylated AMPKα1/2, but they activated different downstream mechanisms (Dai et al., 2015). Although it is still unclear how leptin activates AMPK in our experimental conditions, the adenosine analog AICAR is well established to be up-taken by the cells through adenosine transporters (Gadalla et al., 2004) and phosphorylated by intracellular adenosine kinase into ZMP which binds to AMPK at the same sites as AMP, but with lower affinity (Corton et al., 1995). The involvement of ZMP in purine nucleotide synthesis might interfere with AMPK-downstream pathway leading to autophagy off-target effects. While all AMPK isoforms were characterized in chickens (Proszkowiec-Weglarz et al., 2006), it is still unknown which catalytic and regulatory subunits are predominant in the studied tissues which might probably explain their differential responsiveness to leptin vs. AICAR. In fact, the expression of AMPK isoforms has been shown to be tissue restricted with distinct functions (Suzuki et al., 2007; Lamia et al., 2009; Oakhill et al., 2010).

In conclusion, the finding of the present study are the first evidence of neuroendocrine upregulation of avian autophagy by leptin. Although AMPK is ubiquitously expressed and although leptin activated Ob-Rb/STAT3 signaling pathway in all studied tissues, its effect on AMPK is tissue-specific. The AMPK-independent downstream signaling cascades employed by leptin to induce autophagy in chicken muscle remains a critical underexplored area for future research. Similarly, how leptin activated the chicken hypothalamic and hepatic AMPK is not known and merits further in depth investigation.

## Author contributions

SD conceived and designed the study. AP, GN, PI, EG, and JF conducted the *in vitro* experiments and analyzed the data. MC conducted the ICV injection and the animal experiment. SD wrote the paper with a critical review by MC, WB, WK, TO, and HM.

### Conflict of interest statement

The authors declare that the research was conducted in the absence of any commercial or financial relationships that could be construed as a potential conflict of interest.

## References

[B1] Adachi H. Takemoto Y. Bungo T. Ohkubo T. (2008). Chicken leptin receptor is functional in activating JAK-STATpathway *in vitro*. J. Endocrinol. 197, 335–342. 10.1677/JOE-08-0098 18434363

[B2] Agrawal S. Gollapudi S. Su H. Gupta S. (2011). Leptin activates human B cells to secrete TNF-alpha, IL-6, and IL-10 via JAK2/STAT3 and p38MAPK/ERK1/2 signaling pathway. J. Clin. Immunol. 31, 472–478. 10.1007/s10875-010-9507-1 21243519 PMC3132280

[B3] Andersson U. Filipsson K. Abbott C. R. Woods A. Smith K. Bloom S. R.. (2004). AMP-activated protein kinase plays a role in the control of food intake. J. Biol. Chem. 279, 12005–12008. 10.1074/jbc.C300557200 14742438

[B4] Antin P. B. Ordahl C. P. (1991). Isolation and characterization of an avian myogenic cell line. Dev. Biol. 143, 111–121. 10.1016/0012-1606(91)90058-B 1985013

[B5] Ashwell C. M. Czerwinski S. M. Brocht D. M. McMurtry J. P. (1999). Hormonal regulation of leptin expression in broiler chickens. Am. J. Physiol. 276, R226– R232. 10.1152/ajpregu.1999.276.1.R226 9887199

[B6] Axe E. L. Walker S. A. Manifava M. Chandra P. Roderick H. L. Habermann A.. (2008). Autophagosome formation from membrane compartments enriched in phosphatidylinositol 3-phosphate and dynamically connected to the endoplasmic reticulum. J. Cell Biol. 182, 685–701. 10.1083/jcb.200803137 18725538 PMC2518708

[B7] Bado A. Levasseur S. Attoub S. Kermorgant S. Laigneau J. P. Bortoluzzi M. N.. (1998). The stomach is a source of leptin. Nature 394, 790–793. 9723619 10.1038/29547

[B8] Barth S. Glick D. Macleod K. F. (2010). Autophagy: assays and artifacts. J. Pathol. 221, 117–124. 10.1002/path.2694 20225337 PMC2989884

[B9] Baumann K. (2015). Autophagy: mitophagy receptors unravelled. Nat. Rev. Mol. Cell Biol. 16:580 10.1038/nrm4058 26350072

[B10] Boya P. Reggiori F. Codogno P. (2013). Emerging regulation and functions of autophagy. Nat. Cell Biol. 15, 713–720. 10.1038/ncb2788 23817233 PMC7097732

[B11] Cassano S. Pucino V. La Rocca C. Procaccini C. De Rosa V. Marone G.. (2014). Leptin modulates autophagy in human CD4+CD25- conventional T cells. Metab. Clin. Exp. 63, 1272–1279. 10.1016/j.metabol.2014.06.010 25060689 PMC4180014

[B12] Cinque L. Forrester A. Bartolomeo R. Svelto M. Venditti R. Montefusco S.. (2015). FGF signalling regulates bone growth through autophagy. Nature 528, 272–275. 10.1038/nature16063 26595272

[B13] Corton J. M. Gillespie J. G. Hawley S. A. Hardie D. G. (1995). 5-aminoimidazole-4-carboxamide ribonucleoside. A specific method for activating AMP-activated protein kinase in intact cells? Eur. J. Biochem. 229 558–565. 7744080 10.1111/j.1432-1033.1995.tb20498.x

[B14] Criollo A. Senovilla L. Authier H. Maiuri M. C. Morselli E. Vitale I.. (2010). The IKK complex contributes to the induction of autophagy. EMBO J. 29, 619–631. 10.1038/emboj.2009.364 19959994 PMC2830700

[B15] Cuervo A. M. (2011). Chaperone-mediated autophagy: dice's 'wild' idea about lysosomal selectivity. Nat. Rev. Mol. Cell Biol. 12, 535–541. 10.1038/nrm3150 21750569

[B16] Cui H. Cai F. Belsham D. D. (2006). Leptin signaling in neurotensin neurons involves STAT, MAP kinases ERK1/2, and p38 through c-Fos and ATF1. FASEB J. 20, 2654–2656. 10.1096/fj.06-5989fje 17077290

[B17] Dai Y. L. Huang S. L. Leng Y. (2015). AICAR and metformin exert AMPK-dependent effects on INS-1E pancreatic beta-cell apoptosis via differential downstream mechanisms. Int. J. Biol. Sci. 11, 1272–1280. 10.7150/ijbs.12108 26435693 PMC4582151

[B18] Davis J. L. Masuoka D. T. Gerbrandt L. K. Cherkin A. (1979). Autoradiographic distribution of L-proline in chicks after intracerebral injection. Physiol. Behav. 22, 693–695. 10.1016/0031-9384(79)90233-6 482410

[B19] Denbow D. M. Meade S. Robertson A. McMurtry J. P. Richards M. Ashwell C. (2000). Leptin-induced decrease in food intake in chickens. Physiol. Behav. 69, 359–362. 10.1016/S0031-9384(99)00258-9 10869603

[B20] Deretic V. Levine B. (2009). Autophagy, immunity, and microbial adaptations. Cell Host Microbe 5, 527–549. 10.1016/j.chom.2009.05.016 19527881 PMC2720763

[B21] Dridi S. Hirano Y. Tarallo V. Kim Y. Fowler B. J. Ambati B. K.. (2012). ERK1/2 activation is a therapeutic target in age-related macular degeneration. Proc. Natl. Acad. Sci. U.S.A. 109, 13781–13786. 10.1073/pnas.1206494109 22869729 PMC3427082

[B22] Dridi S. Raver N. Gussakovsky E. E. Derouet M. Picard M. Gertler A.. (2000). Biological activities of recombinant chicken leptin C4S analog compared with unmodified leptins. Am. J. Physiol. Endocrinol. Metab. 279, E116–E123. 10.1152/ajpendo.2000.279.1.E116 10893330

[B23] Farré J. C. Subramani S. (2016). Mechanistic insights into selective autophagy pathways: lessons from yeast. Nat. Rev. Mol. Cell Biol. 17, 537–552. 10.1038/nrm.2016.74 27381245 PMC5549613

[B24] Feng Y. Backues S. K. Baba M. Heo J. M. Harper J. W. Klionsky D. J. (2016). Phosphorylation of Atg9 regulates movement to the phagophore assembly site and the rate of autophagosome formation. Autophagy 12, 648–658. 10.1080/15548627.2016.1157237 27050455 PMC4835963

[B25] Feng Y. Yao Z. Klionsky D. J. (2015). How to control self-digestion: transcriptional, post-transcriptional, and post-translational regulation of autophagy. Trends Cell Biol. 25, 354–363. 10.1016/j.tcb.2015.02.002 25759175 PMC4441840

[B26] Fiorini C. Menegazzi M. Padroni C. Dando I. Dalla Pozza E. Gregorelli A.. (2013). Autophagy induced by p53-reactivating molecules protects pancreatic cancer cells from apoptosis. Apoptosis 18, 337–346. 10.1007/s10495-012-0790-6 23238993

[B27] Friedman J. M. Halaas J. L. (1998). Leptin and the regulation of body weight in mammals. Nature 395, 763–770. 10.1038/27376 9796811

[B28] Fritzen A. M. Madsen A. B. Kleinert M. Treebak J. T. Lundsgaard A. M. Jensen T. E.. (2016). Regulation of autophagy in human skeletal muscle: effects of exercise, exercise training and insulin stimulation. J. Physiol. 594, 745–761. 10.1113/JP271405 26614120 PMC5341711

[B29] Gadalla A. E. Pearson T. Currie A. J. Dale N. Hawley S. A. Sheehan M. (2004). AICA riboside both activates AMP-activated protein kinase and competes with adenosine for the nucleoside transporter in the CA1 region of the rat hippocampus. J. Neurochem. 88, 1272–1282. 10.1046/j.1471-4159.2003.02253.x 15009683

[B30] García-Prat L. Martínez-Vicente M. Perdiguero E. Ortet L. Rodriguez-Ubreva J. Rebollo E.. (2016). Autophagy maintains stemness by preventing senescence. Nature 529, 37–42. 10.1038/nature16187 26738589

[B31] Grotemeier A. Alers S. Pfisterer S. G. Paasch F. Daubrawa M. Dieterle A.. (2010). AMPK-independent induction of autophagy by cytosolic Ca2+ increase. Cell. Signal. 22, 914–925. 10.1016/j.cellsig.2010.01.015 20114074

[B32] Gutierrez M. G. Munafó D. B. Berón W. Colombo M. I. (2004). Rab7 is required for the normal progression of the autophagic pathway in mammalian cells. J. Cell Sci. 117, 2687–2697. 10.1242/jcs.01114 15138286

[B33] Hailey D. W. Rambold A. S. Satpute-Krishnan P. Mitra K. Sougrat R. Kim P. K.. (2010). Mitochondria supply membranes for autophagosome biogenesis during starvation. Cell 141, 656–667. 10.1016/j.cell.2010.04.009 20478256 PMC3059894

[B34] Hamer G. Matilainen O. Holmberg C. I. (2010). A photoconvertible reporter of the ubiquitin-proteasome system *in vivo*. Nat. Methods 7, 473–478. 10.1038/nmeth.1460 20453865

[B35] Hara T. Nakamura K. Matsui M. Yamamoto A. Nakahara Y. Suzuki-Migishima R.. (2006). Suppression of basal autophagy in neural cells causes neurodegenerative disease in mice. Nature 441, 885–889. 10.1038/nature04724 16625204

[B36] Hardie D. G. (2007). AMP-activated/SNF1 protein kinases: conserved guardians of cellular energy. Nat. Rev. Mol. Cell Biol. 8, 774–785. 10.1038/nrm2249 17712357

[B37] He C. Bassik M. C. Moresi V. Sun K. Wei Y. Zou Z.. (2012). Exercise-induced BCL2-regulated autophagy is required for muscle glucose homeostasis. Nature 481, 511–515. 10.1038/nature10758 22258505 PMC3518436

[B38] Hoggard N. Hunter L. Duncan J. S. Williams L. M. Trayhurn P. Mercer J. G. (1997a). Leptin and leptin receptor mRNA and protein expression in the murine fetus and placenta. Proc. Natl. Acad. Sci. U.S.A. 94, 11073–11078. 9380761 10.1073/pnas.94.20.11073 PMC23608

[B39] Hoggard N. Mercer J. G. Rayner D. V. Moar K. Trayhurn P. Williams L. M. (1997b). Localization of leptin receptor mRNA splice variants in murine peripheral tissues by RT-PCR and *in situ* hybridization. Biochem. Biophys. Res. Commun. 232, 383–387. 9125186 10.1006/bbrc.1997.6245

[B40] Horev G. Einat P. Aharoni T. Eshdat Y. Friedman-Einat M. (2000). Molecular cloning and properties of the chicken leptin-receptor. (CLEPR) gene. Mol. Cell. Endocrinol. 162, 95–106. 10.1016/S0303-7207(00)00205-7 10854702

[B41] Ihle J. N. (1995). Cytokine receptor signalling. Nature 377, 591–594. 10.1038/377591a0 7566171

[B42] Itakura E. Kishi-Itakura C. Mizushima N. (2012). The hairpin-type tail-anchored SNARE syntaxin 17 targets to autophagosomes for fusion with endosomes/lysosomes. Cell 151, 1256–1269. 10.1016/j.cell.2012.11.001 23217709

[B43] Jäger S. Bucci C. Tanida I. Ueno T. Kominami E. Saftig P.. (2004). Role for Rab7 in maturation of late autophagic vacuoles. J. Cell Sci. 117, 4837–4848. 10.1242/jcs.01370 15340014

[B44] Jinno-Oue A. Shimizu N. Hamada N. Wada S. Tanaka A. Shinagawa M.. (2010). Irradiation with carbon ion beams induces apoptosis, autophagy, and cellular senescence in a human glioma-derived cell line. Int. J. Radiat. Oncol. Biol. Phys. 76, 229–241. 10.1016/j.ijrobp.2009.08.054 20005456

[B45] Kabeya Y. Mizushima N. Ueno T. Yamamoto A. Kirisako T. Noda T.. (2000). LC3, a mammalian homologue of yeast Apg8p, is localized in autophagosome membranes after processing. EMBO J. 19, 5720–5728. 10.1093/emboj/19.21.5720 11060023 PMC305793

[B46] Kaneko H. Dridi S. Tarallo V. Gelfand B. D. Fowler B. J. Cho W. G.. (2011). DICER1 deficit induces Alu RNA toxicity in age-related macular degeneration. Nature 471, 325–330. 10.1038/nature09830 21297615 PMC3077055

[B47] Kaufmann A. Beier V. Franquelim H. G. Wollert T. (2014). Molecular mechanism of autophagic membrane-scaffold assembly and disassembly. Cell 156, 469–481. 10.1016/j.cell.2013.12.022 24485455

[B48] Khaminets A. Heinrich T. Mari M. Grumati P. Huebner A. K. Akutsu M.. (2015). Regulation of endoplasmic reticulum turnover by selective autophagy. Nature 522, 354–358. 10.1038/nature14498 26040720

[B49] Kielar D. Clark J. S. Ciechanowicz A. Kurzawski G. Sulikowski T. Naruszewicz M. (1998). Leptin receptor isoforms expressed in human adipose tissue. Metab. Clin. Exp. 47, 844–847. 10.1016/S0026-0495(98)90124-X 9667233

[B50] Kimmey J. M. Huynh J. P. Weiss L. A. Park S. Kambal A. Debnath J.. (2015). Unique role for ATG5 in neutrophil-mediated immunopathology during M. tuberculosis infection. Nature 528, 565–569. 10.1038/nature16451 26649827 PMC4842313

[B51] Kimura S. Noda T. Yoshimori T. (2008). Dynein-dependent movement of autophagosomes mediates efficient encounters with lysosomes. Cell Struct. Funct. 33, 109–122. 10.1247/csf.08005 18388399

[B52] Klionsky D. J. Abdelmohsen K. Abe A. Abedin M. J. Abeliovich H. Acevedo Arozena A.. (2016). Guidelines for the use and interpretation of assays for monitoring autophagy. (3rd edition). Autophagy 12, 1–222. 10.1080/15548627.2015.1100356 26799652 PMC4835977

[B53] Kraft C. Deplazes A. Sohrmann M. Peter M. (2008). Mature ribosomes are selectively degraded upon starvation by an autophagy pathway requiring the Ubp3p/Bre5p ubiquitin protease. Nat. Cell Biol. 10, 602–610. 10.1038/ncb1723 18391941

[B54] Kuenzel W. J. Masson M. (1988). A Stereotaxic Atlas of the Brain of the Chick. (Gallus domesticus). Baltimore, MD: Johns Hopkins University Press.

[B55] Kuma A. Hatano M. Matsui M. Yamamoto A. Nakaya H. Yoshimori T.. (2004). The role of autophagy during the early neonatal starvation period. Nature 432, 1032–1036. 10.1038/nature03029 15525940

[B56] Kundu M. Lindsten T. Yang C. Y. Wu J. Zhao F. Zhang J.. (2008). Ulk1 plays a critical role in the autophagic clearance of mitochondria and ribosomes during reticulocyte maturation. Blood 112, 1493–1502. 10.1182/blood-2008-02-137398 18539900 PMC2515143

[B57] Lamia K. A. Sachdeva U. M. DiTacchio L. Williams E. C. Alvarez J. G. Egan D. F.. (2009). AMPK regulates the circadian clock by cryptochrome phosphorylation and degradation. Science 326, 437–440. 10.1126/science.1172156 19833968 PMC2819106

[B58] Lassiter K. Greene E. Piekarski A. Faulkner O. B. Hargis B. M. Bottje W.. (2015). Orexin system is expressed in avian muscle cells and regulates mitochondrial dynamics. Am. J. Physiol. Regul. Integr. Comp. Physiol. 308, R173–R187. 10.1152/ajpregu.00394.2014 25502749

[B59] Lee J. A. Beigneux A. Ahmad S. T. Young S. G. Gao F. B. (2007). ESCRT-III dysfunction causes autophagosome accumulation and neurodegeneration. Curr. Biol. 17, 1561–1567. 10.1016/j.cub.2007.07.029 17683935

[B60] Levine B. Klionsky D. J. (2004). Development by self-digestion: molecular mechanisms and biological functions of autophagy. Dev. Cell 6, 463–477. 10.1016/S1534-5807(04)00099-1 15068787

[B61] Levine B. Kroemer G. (2008). Autophagy in the pathogenesis of disease. Cell 132, 27–42. 10.1016/j.cell.2007.12.018 18191218 PMC2696814

[B62] Levine B. Mizushima N. Virgin H. W. (2011). Autophagy in immunity and inflammation. Nature 469, 323–335. 10.1038/nature09782 21248839 PMC3131688

[B63] Liang C. Lee J. S. Inn K. S. Gack M. U. Li Q. Roberts E. A.. (2008). Beclin1-binding UVRAG targets the class C Vps complex to coordinate autophagosome maturation and endocytic trafficking. Nat. Cell Biol. 10, 776–787. 10.1038/ncb1740 18552835 PMC2878716

[B64] Lõhmus M. Olin M. Sundstrom L. F. Troedsson M. H. Molitor T. W. El Halawani M. (2004). Leptin increases T-cell immune response in birds. Gen. Comp. Endocrinol. 139, 245–250. 10.1016/j.ygcen.2004.09.011 15560871

[B65] Lohmus M. Sundstrom L. F. El Halawani M. Silverin B. (2003). Leptin depresses food intake in great tits. (Parus major). Gen. Comp. Endocrinol. 131, 57–61. 10.1016/S0016-6480(02)00643-3 12620247

[B66] Lord G. M. Matarese G. Howard J. K. Baker R. J. Bloom S. R. Lechler R. I. (1998). Leptin modulates the T-cell immune response and reverses starvation-induced immunosuppression. Nature 394, 897–901. 10.1038/29795 9732873

[B67] Maier H. J. Cottam E. M. Stevenson-Leggett P. Wilkinson J. A. Harte C. J. Wileman T.. (2013). Visualizing the autophagy pathway in avian cells and its application to studying infectious bronchitis virus. Autophagy 9, 496–509. 10.4161/auto.23465 23328491 PMC3627666

[B68] Malik S. A. Mariño G. BenYounès A. Shen S. Harper F. Maiuri M. C.. (2011). Neuroendocrine regulation of autophagy by leptin. Cell Cycle 10, 2917–2923. 10.4161/cc.10.17.17067 21857156

[B69] Manjunathan R. Ragunathan M. (2015). In ovo administration of human recombinant leptin shows dose dependent angiogenic effect on chicken chorioallantoic membrane. Biol. Res. 48:29 10.1186/s40659-015-0021-z 26060038 PMC4470073

[B70] Mao K. Chew L. H. Inoue-Aono Y. Cheong H. Nair U. Popelka H.. (2013). Atg29 phosphorylation regulates coordination of the Atg17-Atg31-Atg29 complex with the Atg11 scaffold during autophagy initiation. Proc. Natl. Acad. Sci. U.S.A. 110 E2875–E2884. 10.1073/pnas.1300064110 23858448 PMC3732952

[B71] Marwarha G. Raza S. Meiers C. Ghribi O. (2014). Leptin attenuates BACE1 expression and amyloid-beta genesis via the activation of SIRT1 signaling pathway. Biochim. Biophys. Acta 1842, 1587–1595. 10.1016/j.bbadis.2014.05.015 24874077 PMC4125440

[B72] Minokoshi Y. Alquier T. Furukawa N. Kim Y. B. Lee A. Xue B.. (2004). AMP-kinase regulates food intake by responding to hormonal and nutrient signals in the hypothalamus. Nature 428, 569–574. 10.1038/nature02440 15058305

[B73] Minokoshi Y. Kim Y. B. Peroni O. D. Fryer L. G. Müller C. Carling D.. (2002). Leptin stimulates fatty-acid oxidation by activating AMP-activated protein kinase. Nature 415, 339–343. 10.1038/415339a 11797013

[B74] Morselli E. Maiuri M. C. Markaki M. Megalou E. Pasparaki A. Palikaras K.. (2010). Caloric restriction and resveratrol promote longevity through the Sirtuin-1-dependent induction of autophagy. Cell Death Dis. 1:e10. 10.1038/cddis.2009.8 21364612 PMC3032517

[B75] Mortimore G. E. Lardeux B. R. Adams C. E. (1988). Regulation of microautophagy and basal protein turnover in rat liver. Effects of short-term starvation. J. Biol. Chem. 263, 2506–2512. 3257493

[B76] Nagarajan G. Jurkevich A. Kang S. W. Kuenzel W. J. (2017). Anatomical and functional implications of corticotrophin-releasing hormone neurones in a septal nucleus of the avian brain: an emphasis on glial-neuronal interaction via V1a receptors *in vitro*. J. Neuroendocrinol. 29, 10.1111/jne.12494 28614607

[B77] Nair U. Jotwani A. Geng J. Gammoh N. Richerson D. Yen W. L.. (2011). SNARE proteins are required for macroautophagy. Cell 146, 290–302. 10.1016/j.cell.2011.06.022 21784249 PMC3143362

[B78] Nemazanyy I. Montagnac G. Russell R. C. Morzyglod L. Burnol A. F. Guan K. L.. (2015). Class III PI3K regulates organismal glucose homeostasis by providing negative feedback on hepatic insulin signalling. Nat. Commun. 6:8283. 10.1038/ncomms9283 26387534 PMC4579570

[B79] Nepal S. Kim M. J. Hong J. T. Kim S. H. Sohn D. H. Lee S. H.. (2015). Autophagy induction by leptin contributes to suppression of apoptosis in cancer cells and xenograft model: involvement of p53/FoxO3A axis. Oncotarget 6, 7166–7181. 10.18632/oncotarget.3347 25704884 PMC4466676

[B80] Nguyen P. Greene E. Ishola P. Huff G. Donoghue A. Bottje W.. (2015). Chronic mild cold conditioning modulates the expression of hypothalamic neuropeptide and intermediary metabolic-related genes and improves growth performances in young chicks. PLoS ONE 10:e0142319. 10.1371/journal.pone.0142319 26569484 PMC4646505

[B81] Nishida Y. Arakawa S. Fujitani K. Yamaguchi H. Mizuta T. Kanaseki T.. (2009). Discovery of Atg5/Atg7-independent alternative macroautophagy. Nature 461, 654–658. 10.1038/nature08455 19794493

[B82] Niswender K. D. Morton G. J. Stearns W. H. Rhodes C. J. Myers M. G. Jr. Schwartz M. W. (2001). Intracellular signalling. key enzyme in leptin-induced anorexia. Nature 413 794–795. 10.1038/35101657 11677594

[B83] Oakhill J. S. Chen Z. P. Scott J. W. Steel R. Castelli L. A. Ling N. (2010). β-Subunit myristoylation is the gatekeeper for initiating metabolic stress sensing by AMP-activated protein kinase. (AMPK). Proc. Natl. Acad. Sci. U.S.A. 107, 19237–19241. 10.1073/pnas.1009705107 20974912 PMC2984171

[B84] Ohkubo T. Hirota K. Murase D. Adachi H. Nozawa-Takeda T. Sugita S. (2014). Avian blood induced intranuclear translocation of STAT3 via the chicken leptin receptor. Comp. Biochem. Physiol. B. Biochem. Mol. Biol. 174, 9–14. 10.1016/j.cbpb.2014.05.001 24858374

[B85] Ohkubo T. Tanaka M. Nakashima K. (2000). Structure and tissue distribution of chicken leptin receptor. (cOb-R) mRNA. Biochim. Biophys. Acta 1491 303–308. 10.1016/S0167-4781(00)00046-4 10760595

[B86] Ohsumi Y. (2014). Historical landmarks of autophagy research. Cell Res. 24, 9–23. 10.1038/cr.2013.169 24366340 PMC3879711

[B87] Oka T. Hikoso S. Yamaguchi O. Taneike M. Takeda T. Tamai T.. (2012). Mitochondrial DNA that escapes from autophagy causes inflammation and heart failure. Nature 485, 251–255. 10.1038/nature10992 22535248 PMC3378041

[B88] Orenstein S. J. Kuo S. H. Tasset I. Arias E. Koga H. Fernandez-Carasa I.. (2013). Interplay of LRRK2 with chaperone-mediated autophagy. Nat. Neurosci. 16, 394–406. 10.1038/nn.3350 23455607 PMC3609872

[B89] Paglin S. Hollister T. Delohery T. Hackett N. McMahill M. Sphicas E.. (2001). A novel response of cancer cells to radiation involves autophagy and formation of acidic vesicles. Cancer Res. 61, 439–444. 11212227

[B90] Palikaras K. Lionaki E. Tavernarakis N. (2015). Coordination of mitophagy and mitochondrial biogenesis during ageing in C. elegans. Nature 521, 525–528. 10.1038/nature14300 25896323

[B91] Piekarski A. Decuypere E. Buyse J. Dridi S. (2016). Chenodeoxycholic acid reduces feed intake and modulates the expression of hypothalamic neuropeptides and hepatic lipogenic genes in broiler chickens. Gen. Comp. Endocrinol. 229, 74–83. 10.1016/j.ygcen.2016.03.007 26965947

[B92] Piekarski A. Khaldi S. Greene E. Lassiter K. Mason J. G. Anthony N.. (2014a). Tissue distribution, gender- and genotype-dependent expression of autophagy-related genes in avian species. PLoS ONE 9:e112449. 10.1371/journal.pone.0112449 25386921 PMC4227737

[B93] Piekarski A. L. Kong B. W. Lassiter K. Hargis B. M. Bottje W. G. (2014b). Cell bioenergetics in Leghorn male hepatoma cells and immortalized chicken liver cells in response to 4-hydroxy 2-nonenal-induced oxidative stress. Poult. Sci. 93, 2870–2877. 10.3382/ps.2014-04113 25143593

[B94] Pimkina J. Murphy M. E. (2009). ARF, autophagy and tumor suppression. Autophagy 5, 397–399. 10.4161/auto.5.3.7782 19221462 PMC2667648

[B95] Proszkowiec-Weglarz M. Richards M. P. Ramachandran R. McMurtry J. P. (2006). Characterization of the AMP-activated protein kinase pathway in chickens. Comp. Biochem. Physiol. B. Biochem. Mol. Biol. 143, 92–106. 10.1016/j.cbpb.2005.10.009 16343965

[B96] Ravikumar B. Acevedo-Arozena A. Imarisio S. Berger Z. Vacher C. O'Kane C. J.. (2005). Dynein mutations impair autophagic clearance of aggregate-prone proteins. Nat. Genet. 37, 771–776. 10.1038/ng1591 15980862

[B97] Rosenfeldt M. T. O'Prey J. Morton J. P. Nixon C. MacKay G. Mrowinska A.. (2013). p53 status determines the role of autophagy in pancreatic tumour development. Nature 504, 296–300. 10.1038/nature12865 24305049

[B98] Scherz-Shouval R. Shvets E. Fass E. Shorer H. Gil L. Elazar Z. (2007). Reactive oxygen species are essential for autophagy and specifically regulate the activity of Atg4. EMBO J. 26, 1749–1760. 10.1038/sj.emboj.7601623 17347651 PMC1847657

[B99] Schmittgen T. D. Livak K. J. (2008). Analyzing real-time PCR data by the comparative C(T) method. Nat. Protoc. 3, 1101–1108. 10.1038/nprot.2008.73 18546601

[B100] Scott M. M. Lachey J. L. Sternson S. M. Lee C. E. Elias C. F. Friedman J. M.. (2009). Leptin targets in the mouse brain. J. Comp. Neurol. 514, 518–532. 10.1002/cne.22025 19350671 PMC2710238

[B101] Seroussi E. Cinnamon Y. Yosefi S. Genin O. Smith J. G. Rafati N.. (2016). Identification of the long-sought leptin in chicken and duck: expression pattern of the highly GC-rich avian leptin fits an autocrine/paracrine rather than endocrine function. Endocrinology 157, 737–751. 10.1210/en.2015-1634 26587783

[B102] Singh R. Kaushik S. Wang Y. Xiang Y. Novak I. Komatsu M.. (2009). Autophagy regulates lipid metabolism. Nature 458, 1131–1135. 10.1038/nature07976 19339967 PMC2676208

[B103] Słupecka M. Wolinski J. Gajewska M. Pierzynowski S. G. (2014). Enteral leptin administration affects intestinal autophagy in suckling piglets. Domest. Anim. Endocrinol. 46, 12–19. 10.1016/j.domaniend.2013.09.007 24135555

[B104] Sun Z. Dragon S. Becker A. Gounni A. S. (2013). Leptin inhibits neutrophil apoptosis in children via ERK/NF-kappaB-dependent pathways. PLoS ONE 8:e55249. 10.1371/journal.pone.0055249 23383125 PMC3561393

[B105] Suzuki A. Okamoto S. Lee S. Saito K. Shiuchi T. Minokoshi Y. (2007). Leptin stimulates fatty acid oxidation and peroxisome proliferator-activated receptor alpha gene expression in mouse C2C12 myoblasts by changing the subcellular localization of the alpha2 form of AMP-activated protein kinase. Mol. Cell. Biol. 27, 4317–4327. 10.1128/MCB.02222-06 17420279 PMC1900064

[B106] Sweeney G. (2002). Leptin signalling. Cell. Signal. 14, 655–663. 10.1016/S0898-6568(02)00006-2 12020765

[B107] Takahashi Y. Okimura Y. Mizuno I. Iida K. Takahashi T. Kaji H.. (1997). Leptin induces mitogen-activated protein kinase-dependent proliferation of C3H10T1/2 cells. J. Biol. Chem. 272, 12897–12900. 10.1074/jbc.272.20.12897 9148892

[B108] Tanaka Y. Guhde G. Suter A. Eskelinen E. L. Hartmann D. Lüllmann-Rauch R.. (2000). Accumulation of autophagic vacuoles and cardiomyopathy in LAMP-2-deficient mice. Nature 406 902–906. 10.1038/35022595 10972293

[B109] Taouis M. Chen J. W. Daviaud C. Dupont J. Derouet M. Simon J. (1998). Cloning the chicken leptin gene. Gene 208, 239–242. 10.1016/S0378-1119(97)00670-7 9524275

[B110] Todde V. Veenhuis M. van der Klei I. J. (2009). Autophagy: principles and significance in health and disease. Biochim. Biophys. Acta 1792, 3–13. 10.1016/j.bbadis.2008.10.016 19022377

[B111] Tracy K. Velentzas P. D. Baehrecke E. H. (2016). Ral GTPase and the exocyst regulate autophagy in a tissue-specific manner. EMBO Rep. 17, 110–121. 10.15252/embr.201541283 26598552 PMC4718410

[B112] Tudurí E. Bruin J. E. Denroche H. C. Fox J. K. Johnson J. D. Kieffer T. J. (2013). Impaired Ca(2+) signaling in beta-cells lacking leptin receptors by Cre-loxP recombination. PLoS ONE 8:e71075. 10.1371/journal.pone.0071075 23936486 PMC3731269

[B113] Vaisse C. Halaas J. L. Horvath C. M. Darnell J. E. Jr. Stoffel M. Friedman J. M. (1996). Leptin activation of Stat3 in the hypothalamus of wild-type and ob/ob mice but not db/db mice. Nat. Genet. 14, 95–97. 10.1038/ng0996-95 8782827

[B114] Vernooy J. H. Drummen N. E. van Suylen R. J. Cloots R. H. Möller G. M. Bracke K. R.. (2009). Enhanced pulmonary leptin expression in patients with severe COPD and asymptomatic smokers. Thorax 64, 26–32. 10.1136/thx.2007.085423 18835960

[B115] Wang J. Zhou J. Y. Kho D. Reiners J. J. Jr. Wu G. S. (2016). Role for DUSP1. (dual-specificity protein phosphatase 1) in the regulation of autophagy. Autophagy 12, 1791–1803. 10.1080/15548627.2016.1203483 27459239 PMC5079544

[B116] Wei Y. Pattingre S. Sinha S. Bassik M. Levine B. (2008). JNK1-mediated phosphorylation of Bcl-2 regulates starvation-induced autophagy. Mol. Cell 30, 678–688. 10.1016/j.molcel.2008.06.001 18570871 PMC2478643

[B117] Wiesner G. Vaz M. Collier G. Seals D. Kaye D. Jennings G.. (1999). Leptin is released from the human brain: influence of adiposity and gender. J. Clin. Endocrinol. Metab. 84, 2270–2274. 10.1210/jc.84.7.2270 10404789

[B118] Xie Y. Kang R. Sun X. Zhong M. Huang J. Klionsky D. J.. (2015). Posttranslational modification of autophagy-related proteins in macroautophagy. Autophagy 11, 28–45. 10.4161/15548627.2014.984267 25484070 PMC4502723

[B119] Zamorano P. L. Mahesh V. B. De Sevilla L. M. Chorich L. P. Bhat G. K. Brann D. W. (1997). Expression and localization of the leptin receptor in endocrine and neuroendocrine tissues of the rat. Neuroendocrinology 65, 223–228. 10.1159/000127276 9088004

[B120] Zhang Y. Proenca R. Maffei M. Barone M. Leopold L. Friedman J. M. (1994). Positional cloning of the mouse obese gene and its human homologue. Nature 372, 425–432. 10.1038/372425a0 7984236

