# Supplementary material for: AMP-Activated Protein Kinase Mediates the Effect of Leptin on Avian Autophagy in a Tissue-Specific Manner
Source: Front Physiol. 2018 May 15;9:541. doi: 10.3389/fphys.2018.00541 (PMC5963154; doi:10.3389/fphys.2018.00541)
Supplement: Supplementary file 1 [file Data_Sheet_1.DOCX]

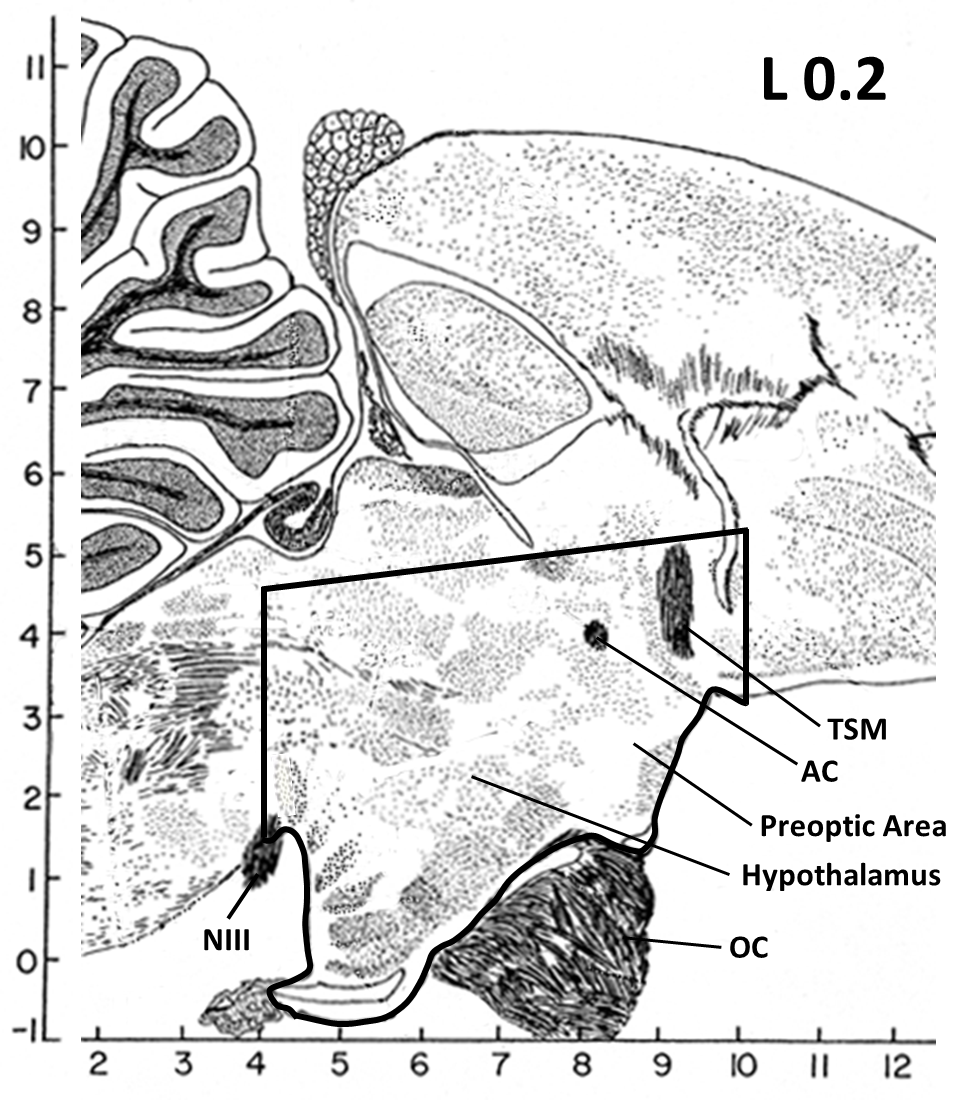


**Figure S1. Sagittal view of a chicken brain near midline (L0.2).** )  The dark line shows the borders of the dissection that included the hypothalamus and preoptic area.  Units of distance on the x and y axis are in mm. The thickness of each dissected piece was 3.0mm (1.5mm on each side of midline).  Landmarks used to make the dissection were four fiber tracts:  AC = anterior commissure, NIII = third cranial nerve, OC = optic chiasma, TSM = septopallio-mesencephalic tract
