# Supplementary material for: AMP-Activated Protein Kinase Mediates the Effect of Leptin on Avian Autophagy in a Tissue-Specific Manner
Source: Front Physiol. 2018 May 15;9:541. doi: 10.3389/fphys.2018.00541 (PMC5963154; doi:10.3389/fphys.2018.00541)
Supplement: Supplementary file 2 [file Data_Sheet_2.DOCX]

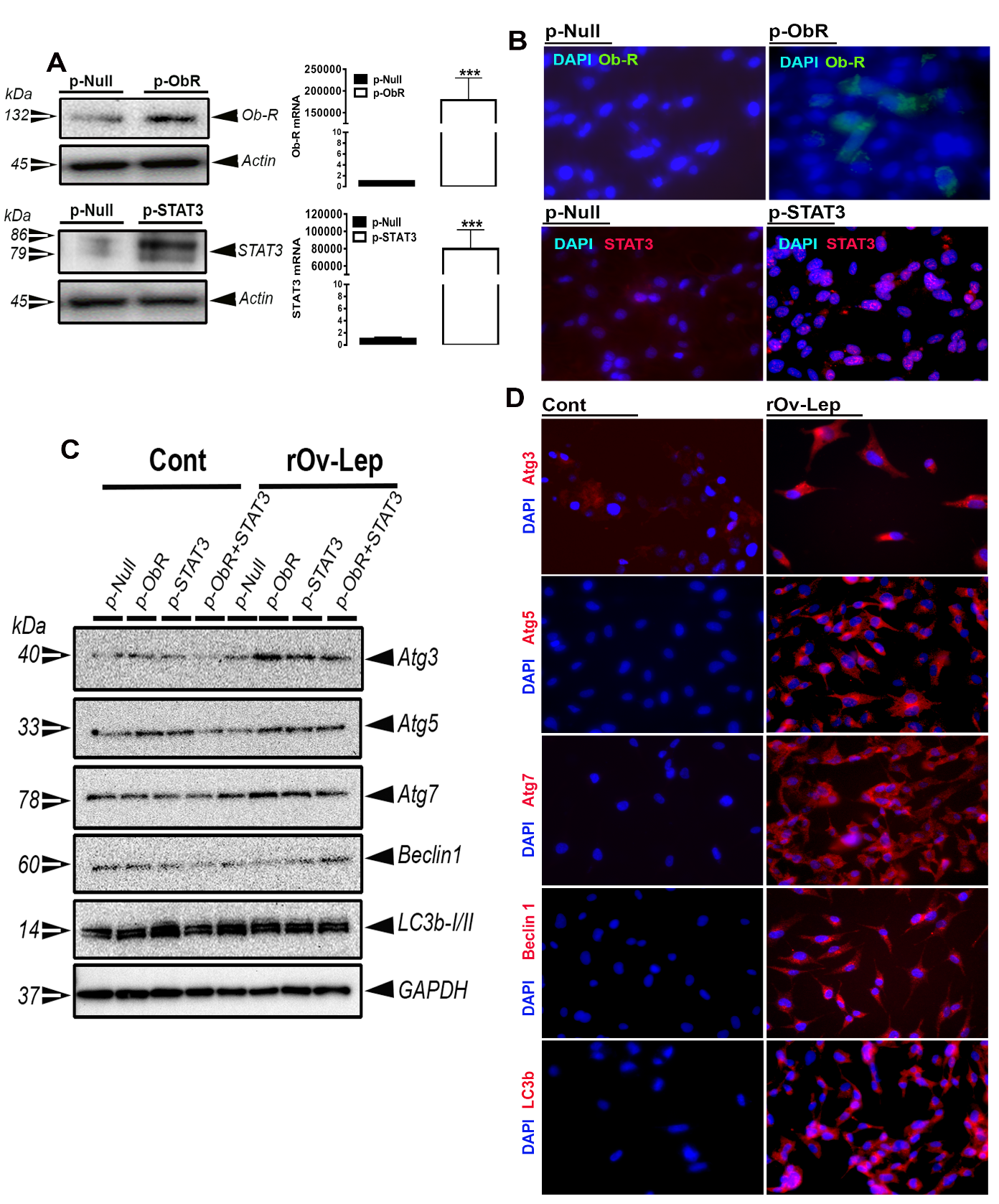


**Figure S2. Effect of leptin treatment on Ob-Rb/STAT pathway and autophagy in Sim-CEL cells overexpressing chicken Ob-Rb and STAT3.** Sim-CEL cells were efficiently co-transfected with chicken Ob-Rb and STAT3 (A, B). Leptin treatment for 24h activates autophagy markers in Sim-CEL cells overexpressing chicken Ob-Rb and STAT3 as illustrated by Western blot analysis (C) and immunofluorescence staining (D). Data are presented as mean ± SEM (representative of 3 experiments). * indicates significant difference at *P*<0.05.
