# Supplementary material for: AMP-Activated Protein Kinase Mediates the Effect of Leptin on Avian Autophagy in a Tissue-Specific Manner
Source: Front Physiol. 2018 May 15;9:541. doi: 10.3389/fphys.2018.00541 (PMC5963154; doi:10.3389/fphys.2018.00541)
Supplement: Supplementary file 4 [file Data_Sheet_4.DOCX]

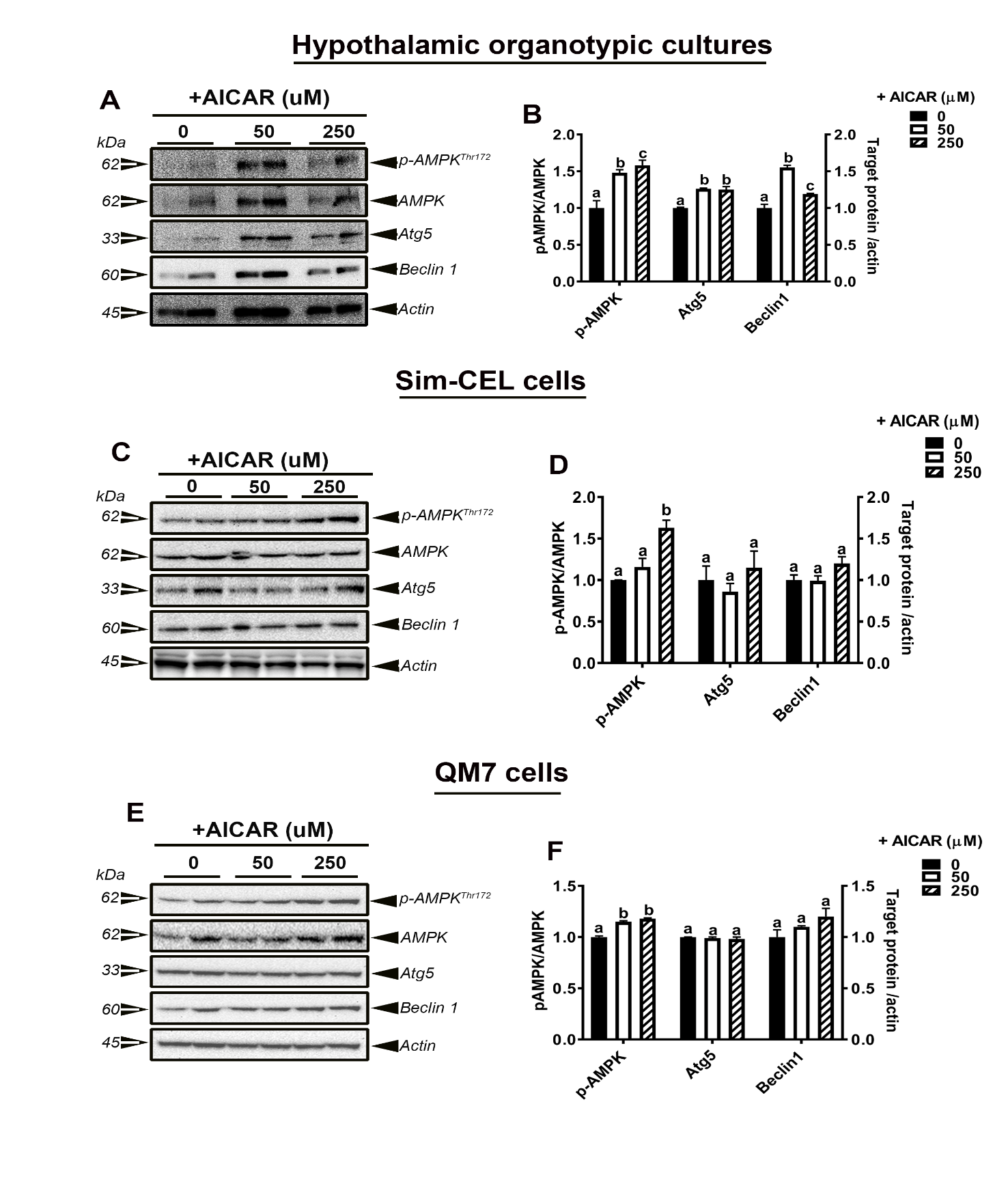


**Figure S4. Effect of AICAR treatment on autophagy in chicken hypothalamic explants and Sim-CEL/QM7 cells.** AICAR treatment activates AMPK in all tissue cultures (A-F), but it induces autophagy-related proteins (Atg5, beclin1) only in the hypothalamic explants (A, B), and not in Sim-CEL (C, D) or QM7 cells (E, F). Data are presented as mean ± SEM (representative of 3 experiments). Different letters indicate indicates significant difference at *P*<0.05.
